# Rare or Unusual Non-Fermenting Gram-Negative Bacteria: Therapeutic Approach and Antibiotic Treatment Options

**DOI:** 10.3390/antibiotics14030306

**Published:** 2025-03-16

**Authors:** Nicholas Geremia, Andrea Marino, Andrea De Vito, Federico Giovagnorio, Stefano Stracquadanio, Agnese Colpani, Stefano Di Bella, Giordano Madeddu, Saverio Giuseppe Parisi, Stefania Stefani, Giuseppe Nunnari

**Affiliations:** 1 Unit of Infectious Diseases, Department of Clinical Medicine, Ospedale “dell’Angelo”, 30174 Venice, Italy; nicholas.geremia@aulss3.veneto.it; 2 Unit of Infectious Diseases, Department of Clinical Medicine, Ospedale Civile “S.S. Giovanni e Paolo”, 30122 Venice, Italy; 3 Unit of Infectious Diseases, Department of Clinical and Experimental Medicine, ARNAS Garibaldi Hospital, University of Catania, 95122 Catania, Italy; giuseppe.nunnari1@unict.it; 4 Unit of Infectious Diseases, Department of Medicine, Surgery and Pharmacy, University of Sassari, 07100 Sassari, Italy; andreadevitoaho@gmail.com (A.D.V.); colpaniagnese@gmail.com (A.C.); giordano@uniss.it (G.M.); 5 Department of Molecular Medicine, University of Padua, 35121 Padua, Italy; federico.giovagnorio@studenti.unipd.it (F.G.); saverio.parisi@unipd.it (S.G.P.); 6 Department of Biomedical and Biotechnological Sciences, University of Catania, 95123 Catania, Italy; s.stracquadanio@unict.it (S.S.); stefania.stefani@unict.it (S.S.); 7 Clinical Department of Medical, Surgical and Health Sciences, Trieste University, 34129 Trieste, Italy; stefano932@gmail.com

**Keywords:** non-fermenting Gram-negative bacteria, non-fermenter bacteria, antimicrobial resistance, rare pathogens, therapeutic strategies, antibiotic treatment

## Abstract

Non-fermenting Gram-negative bacteria (NFGNB) are a heterogeneous group of opportunistic pathogens increasingly associated with healthcare-associated infections. While *Pseudomonas aeruginosa*, *Acinetobacter baumannii,* and *Stenotrophomonas maltophilia* are well known, rarer species such as *Burkholderia cepacia* complex, *Achromobacter* spp., *Chryseobacterium* spp., *Elizabethkingia* spp., *Ralstonia* spp., and others pose emerging therapeutic challenges. Their intrinsic and acquired resistance mechanisms limit effective treatment options, making targeted therapy essential. **Objectives**: This narrative review summarizes the current understanding of rare and unusual NFGNB, their clinical significance, resistance profiles, and evidence-based therapeutic strategies. **Methods**: A literature review was conducted using PubMed, Scopus, and Web of Science to identify relevant studies on the epidemiology, antimicrobial resistance, and treatment approaches to rare NFGNB. **Results**: Rare NFGNB exhibits diverse resistance mechanisms, including β-lactamase production, efflux pumps, and porin modifications. Treatment selection depends on species-specific susceptibility patterns, but some cornerstones can be individuated. Novel β-lactam/β-lactamase inhibitors and combination therapy approaches are being explored for multidrug-resistant isolates. However, clinical data remain limited. **Conclusions**: The increasing incidence of rare NFGNB requires heightened awareness and a tailored therapeutic approach. Given the paucity of clinical guidelines, antimicrobial stewardship and susceptibility-guided treatment are crucial in optimizing patient outcomes.

## 1. Introduction

Non-fermenting Gram-negative bacteria (NFGNB) are a diverse group of aerobic, non-spore-forming bacilli that do not utilize carbohydrates through fermentation [1]. Commonly found in soil and water, they have emerged as significant opportunistic pathogens, particularly in healthcare settings. The most common NFGNB are *Pseudomonas aeruginosa*, *Acinetobacter baumannii*, and species of the genus *Stenotrophomonas* [2]. The pathogenicity of NFGNB is often linked to their intrinsic resistance to multiple antibiotics and their capacity to acquire additional resistance mechanisms [3]. This resistance complicates treatment options and poses substantial challenges in clinical management. For instance, *P. aeruginosa* is known for its antibiotic resistance, making infections difficult to treat [4]. In recent years, infections caused by rare or unusual NFGNB have been increasingly reported, especially among immunocompromised individuals and patients with prolonged hospital stays [5,6,7,8]. These infections are associated with high morbidity and mortality rates, underscoring the need for effective therapeutic strategies [9,10]. Developing new antibiotics and optimizing existing therapeutic approaches are crucial in addressing infections caused by these resistant pathogens [11]. Novel β-lactam/β-lactamase inhibitor (BL/BLI) combinations, such as ceftazidime–avibactam (C/A) and ceftolozane–tazobactam (C/T), have shown promise against certain multidrug-resistant Gram-negative bacteria [12]. Additionally, cefiderocol (FDC), a siderophore cephalosporin, has demonstrated efficacy against a broad spectrum of Gram-negative pathogens, including NFGNB [13,14]. However, most studies focus on *P. aeruginosa*, *A. baumannii*, and *S. maltophilia*; data on rare or unusual NFGNB are limited, making it challenging to develop standardized treatment protocols and fully understand their epidemiology and resistance patterns. Moreover, rare or unusual NFGB comprise a heterogeneous bacteria group with essential differences between different genera and antibiotic spectra activity [15]. This review aims to provide a comprehensive overview of rare or unusual NFGNB, focusing on their taxonomy, microbiological characteristics, and the latest therapeutic approaches, including emerging antibiotic treatment options. Enhancing our understanding of these pathogens and available treatments can improve clinical outcomes and inform future research directions.

## 2. Taxonomy and Microbiology

### 2.1. General Characteristics and Microbiological Diagnosis

The heterogeneous group of NFGNB contains aerobic, non-spore-forming bacilli that do not ferment carbohydrates and derive energy by using simple carbohydrates in an oxidative fashion [1,2].

NFGNB typically grows well on standard culture media, such as blood agar, often appearing without hemolysis [16]. Instead, their growth is enhanced on chocolate agar, particularly with fastidious species like *Burkholderia* and *Stenotrophomonas* [17]. Commercial test systems such as API 20NE, Phoenix, MicroScan, and Vitek 2.0 can easily identify NFGNB. However, misidentification is frequent, and the differentiation of species can be particularly challenging. For example, commercial diagnostic tools can confound *Burkholderia* spp. with *Achromobacter* spp. or *Ralstonia* spp. and, in some cases, may require additional testing for correct microbiological identification [18,19]. Moreover, for some species, such as *Alcaligenes* spp. or *Achromobacter* spp., biochemical identification at the species level is not possible [18]. For these reasons, new microbiological innovations such as 16S rRNA gene sequencing or matrix-assisted laser desorption/ionization–time of flight (MALDI-TOF) are requested to identify some NFGNB [18].

### 2.2. Taxonomy

**Figure 1:**
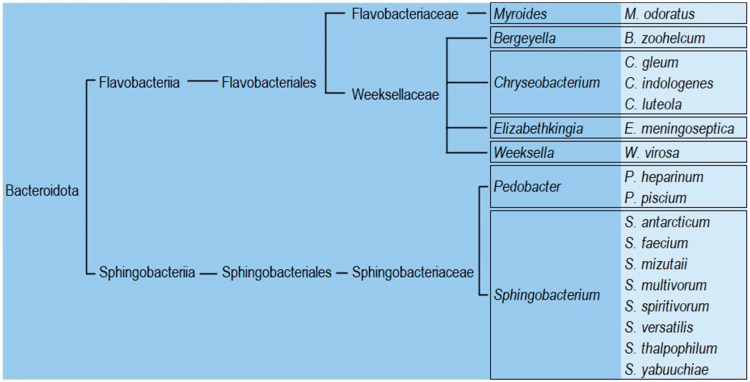
Bacteroidota taxonomical classification of the principal human pathogens.

NFGNB belong to multiple taxonomic groups, primarily the Pseudomonadota phylum and some from the Bacteroidota phylum [18]. The Bacteroidota phylum consists of three main classes, but the two most clinically relevant are Flavobacteriia and Sphingobacteriia [20]. NFGNB Bacteroidota can have a clinical impact, as shown in Figure 1.

The Pseudomonadota phylum is divided into Alphaproteobacteria, Betaproteobacteria, and Gammaproteobacteria, with the last two classes having primary clinical importance [18].

**Figure 2:**
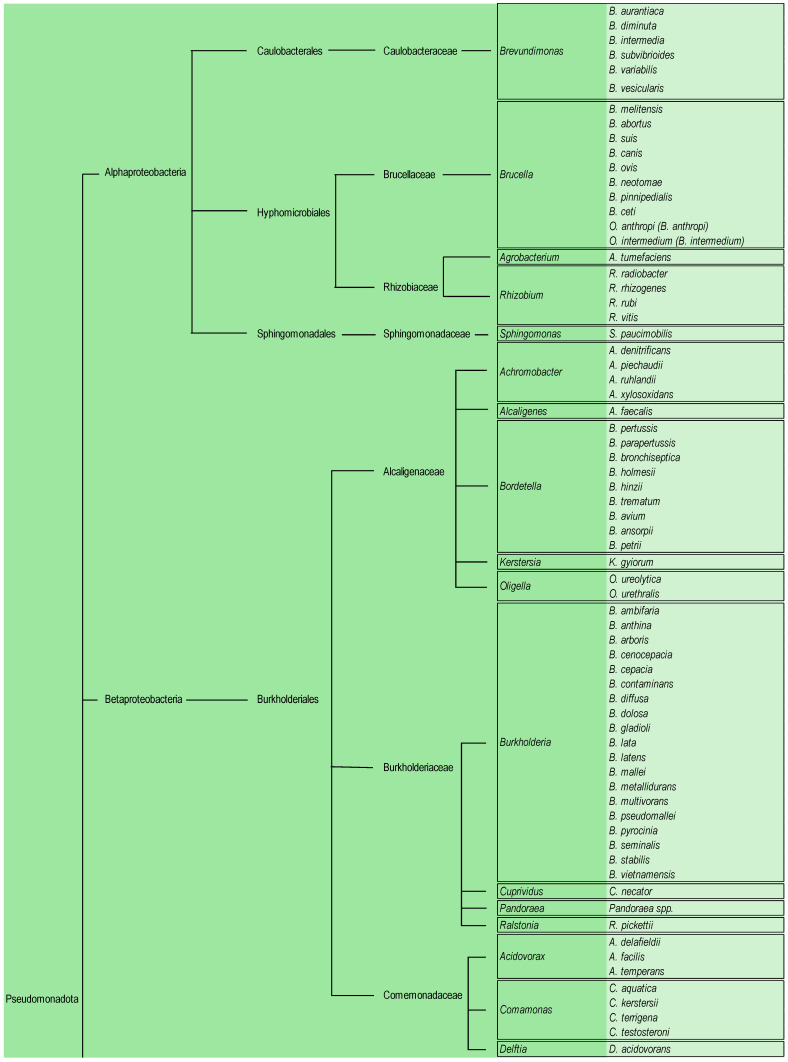

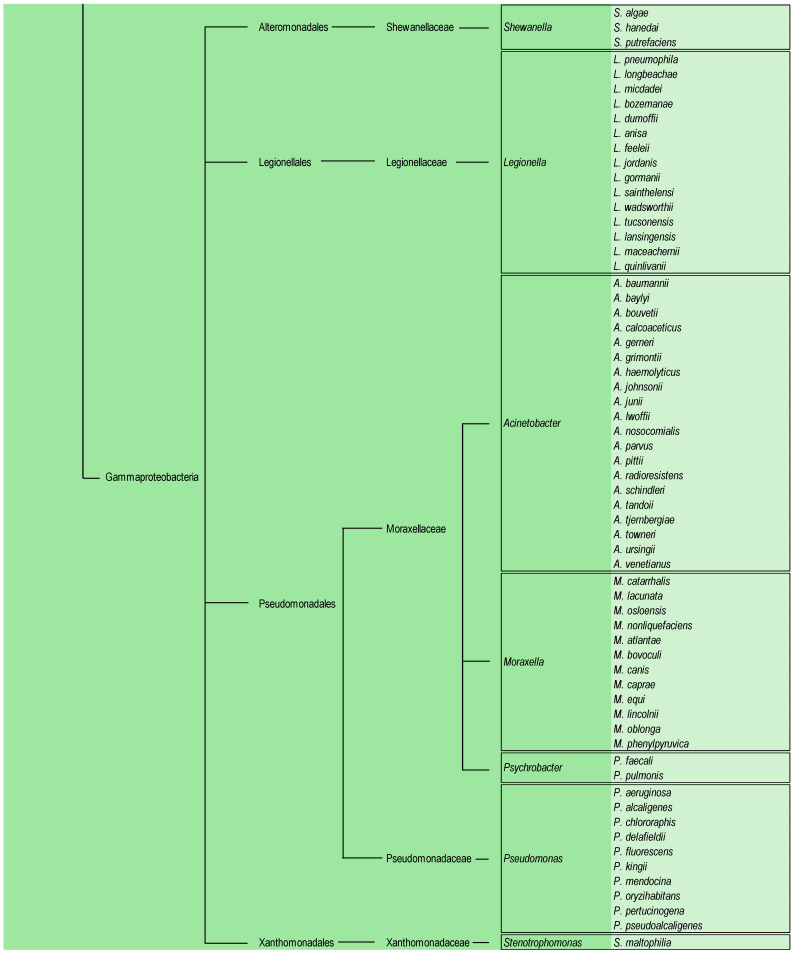
Pseudomonadota taxonomical classification of the principal human pathogens.

Taxonomic complexity and phenotypic similarity represent the most challenging issues for this bacterial group [18]. The widespread use of ribosomal 16S RNA gene sequence analysis has helped to clarify the taxonomic classification of most of these organisms. It has undergone significant taxonomic revisions, with some new species identifications [19]. Pseudomonadota is represented in Figure 2.

### 2.3. Virulence Factors, Role of the Biofilm and General Consideration on Antibiotic Resistance

Several virulence factors are present in NFGNB. Many species had adhesion structures that are fundamental to initiating that process of colonization and biofilm formation. Typically, NFGNB produce fimbriae (or attachment pili), as well as other surface adhesins, such as nonpilus or afimbrial adhesins [21]. Some bacteria can have flagella that mediate motility in viscous media or over surfaces [2]. In NFGNB, the release of toxic compounds (such as exotoxins) and extracellular enzymes that compromise mucosal integrity can reduce the host’s immune system activity and play a role in the infection progression and potentially lead to systemic dissemination [2,4]. Although lipopolysaccharide (LPS), a fundamental compound of the external membrane of Gram-negatives, can also play an essential role in pathogenetic processes relating to local and systemic inflammation [2,4,22]. Specific accessory structures also contribute to the pathogenesis of NFGNB, such as secretion systems, which are utilized by many bacterial pathogens to transport various protein factors (e.g., adhesins, exotoxins, and exoenzymes) onto the bacterial surface, into the surrounding environment, or directly into host cells, disrupting the normal cellular functions and promoting infection [2]. Biofilm is crucial in NFGNB infections; it can permit survival on surfaces and some medical devices, representing a significant risk factor for hospital-acquired infections [23]. Moreover, biofilm significantly impacts antibiotic therapy effectiveness and can lead to immune escape, having a substantial role in the difficulty of NFGNB eradication [2,4,23].

Most NFGNB can survive or even replicate under adverse environmental conditions and can commonly be found in water, soil, plants, vegetables, insects, and other sources [24]. Aerobic NFGNB, other than the most widely described *P. aeruginosa* and *A. baumannii*, can be part of the transient physiologic flora and, in many cases, are not pathogenic for humans [18,19]. However, the rise of immunocompromised conditions in modern medicine has played a significant role in the augmented incidence of these rare bacteria [25].

NFGNB exhibit high intrinsic resistance to major antimicrobial classes, often limiting available treatment options. Both intrinsic and acquired resistance mechanisms have been identified, varying significantly in type and prevalence among different species.

**Figure 3:**
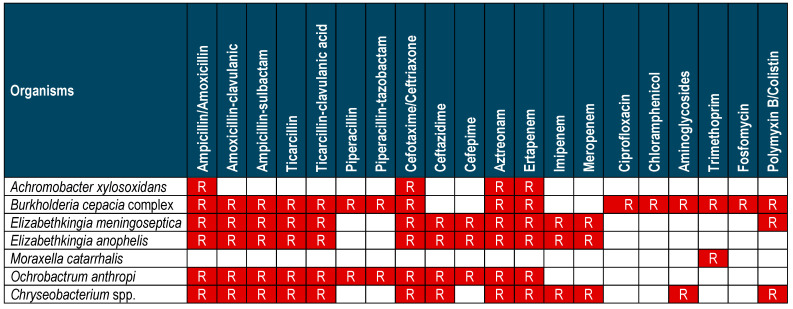
Intrinsic resistance/expected resistant phenotypes in non-fermenter bacteria. R = resistant [27].

Antimicrobial therapy should be based on in vitro antimicrobial susceptibility testing (AST) and, whenever possible, the minimum inhibitory concentrations (MICs) of relevant antimicrobials should be determined [19]. However, no specific breakpoints are available due to the lack of standardized susceptibility testing for several antibiotics in this group of microorganisms [26] and the paucity of clinical data. Moreover, many NFGNB have intrinsic resistant patterns that can be particularly challenging in clinical scenarios. Figure 3 shows the intrinsic resistance of the principal species.

## 3. Achromobacter

First recognized in 1923 by the Committee of the Society of American Bacteriologists, 22 species of the genus Achromobacter have been identified so far, and it is continuously evolving; the most common species worldwide is *A. xylosoxidans* [28,29,30]. The distribution of the other species follows a geographical pattern, with *A. dolens* and *A. insuavis* being the most common in Europe [31]. Genus *Achromobacter* is an obligately aerobic, non-fermentative, oxidase- and catalase-positive, and indole-, urease-, and DNase-negative bacterium [32]. While the identification of the genus *Achromobacte*r has become feasible using MALDI-TOF, allowing us to distinguish it from other non-fermentative bacteria, species identification is still challenging due to the limitations of the MALDI-TOF database and the non-extensive availability of sequencing methods [33].

**Figure 4:**
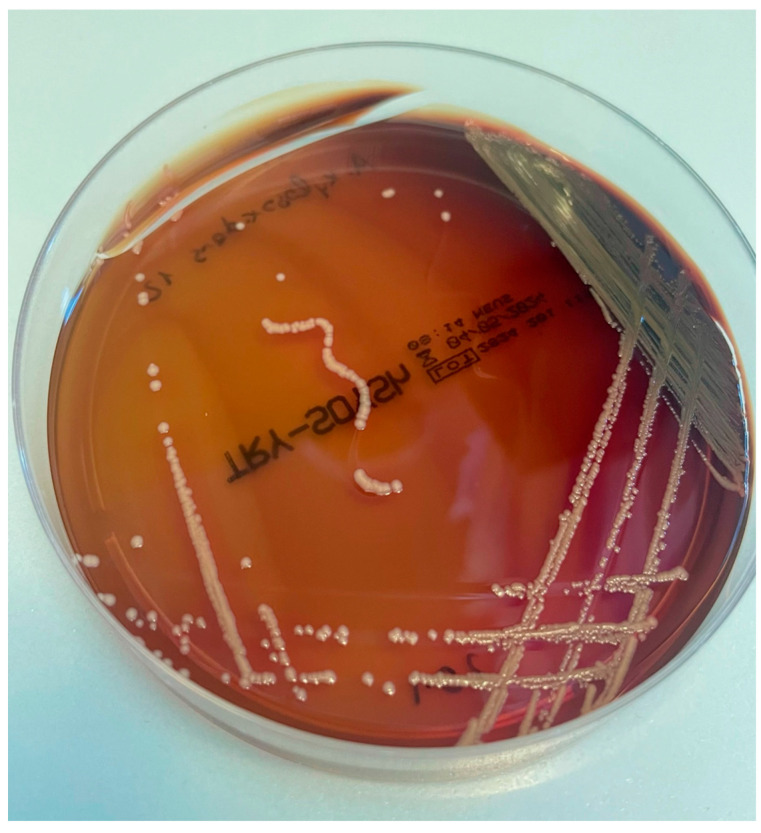
*Achromobacter xylosoxidans* colonies on tryptic soy agar (TSA) with a pale pink to whitish coloration. The colonies are moderately sized, circular, and have a smooth, glistening surface with no evident hemolysis halo.

Although mainly identified from patients with cystic fibrosis, *Achromobacter* spp. infection has also been described in subjects with other underlying conditions, including immunocompetent hosts. Reported cases are mostly hospital-acquired and associated with indwelling catheters. Also, nosocomial outbreaks related to contaminated devices have been described [34,35]. *Achromobacter* can cause pneumonia and various other clinical manifestations [36]; case reports and case series have shown a broad clinical spectrum, suggesting that *Achromobacter* could affect virtually any organ [34,37,38,39,40]. Figure 4 shows *A. xylosoxidans* growing in a Petri dish.

Regarding antimicrobial susceptibility, *Achromobacter* has intrinsic resistance to cephalosporins [except ceftazidime (CAZ)], aztreonam (AZT), ertapenem and aminoglycosides (AMGs); several resistance mechanisms have been described so far. Table 1 synthesizes the principal resistant mechanisms in *Achromobacter* spp.

Among the efflux pumps characterized, AxyABM has been demonstrated to play a crucial role in the extrusion of cephalosporin, AZT, and chloramphenicol [41]. However, it cannot be the sole mechanism underlying antibiotic resistance [43]. As for resistance to AMG, the efflux pump AxyXY-OprZ has been described as a significant determinant of high-level resistance. Also, it accounts for resistance to cefepime (FEP), carbapenems, fluoroquinolones (FQs), tetracyclines (TETs), and erythromycin, despite not being the only resistance determinant [44]. Regarding enzymes, *Achromobacter* spp. constitutively produces OXA-114 β-lactamase, which hydrolyses piperacillin, ticarcillin, benzylpenicillin, and cephalothin; however, the existence of activity against piperacillin, and whether it is affected by tazobactam or not, is not yet clear. The presence of OXA-114 has been proposed as an identification tool for the bacteria. Moreover, *Achromobacter* spp. can acquire other β-lactamases, including VIM and IMP [45]. The Tripoli metallo-β-lactamase (MBL) was first discovered in a strain of *A. xylosoxidans* [46].

Susceptibility breakpoints are not widely accepted; European Committee on Antimicrobial Susceptibility Testing (EUCAST) breakpoints are only given for trimethoprim–sulfamethoxazole (SXT) [47]. At the same time, cephalosporins, imipenem (IMP), ertapenem, AMG, FQ, and colistin (COL) have intrinsically insufficient activity for breakpoints to be determined [47]. In Clinical and Laboratory Standards Institute (CLSI) recommendations for “Other Non-Enterobacterales”, breakpoints are given for piperacillin, P/T, ticarcillin–clavulanate, CAZ, FEP, cefotaxime or ceftriaxone, cefoperazone, moxalactam, AZT, IMP, MEM, gentamicin (GNT), tobramycin, amikacin (AMK), netilmicin, TET, FQ, SXT, and chloramphenicol [48]. As for other promising antibiotics, a study by Beauruelle et al., reporting the in vitro susceptibility testing of 22 antibiotics on *Achromobacter* spp. from patients with cystic fibrosis, showed promising results for IMP (70–91% susceptibility) and FDC (91%) [49].

Despite the increasing data regarding new therapeutic options, the treatment of *Achromobacter* spp. remains challenging, especially in patients with high exposure to multiple antibiotics.

## 4. Alcaligenes

*Alcaligenes* is a genus of Gram-negative, aerobic, non-fermenter, oxidase-positive, catalase-positive, motile rod-shaped bacteria belonging to the family Alcaligenaceae. These bacteria are widely distributed in environmental reservoirs such as soil, water, and organic matter and can colonize the human gastrointestinal tract. Among its species, *Alcaligenes faecalis* is the most clinically relevant, primarily causing opportunistic infections in immunocompromised individuals or patients with underlying health conditions [50]. Although considered a relatively rare pathogen, *A. faecalis* has gained prominence due to its association with healthcare-associated infections and the emergence of significant antimicrobial resistance mechanisms. Its ability to survive in aqueous environments makes it a potential contaminant in hospital settings, particularly in devices such as respirators, hemodialysis systems, and intravenous catheters [51].

A six-year retrospective study in Taiwan reported 61 cases of *A. faecalis* infections between 2014 and 2019, with cystitis being the most frequently observed condition (41%), followed by diabetic foot ulcers (14.8%), pneumonia (13.1%), and bloodstream infections (BSIs) (4.9%) [51]. These infections were frequently polymicrobial, with *A. faecalis* isolated alongside other pathogens, such as *Proteus vulgaris*, *Enterococcus* spp., and *P. aeruginosa*. The majority of infections occurred in elderly patients with comorbidities, particularly those with a history of intravenous antibiotic exposure within three months of diagnosis. *A. faecalis* has also been isolated from surgical wounds, prosthetic devices, and respiratory secretions, reinforcing its role as a significant opportunistic pathogen [52]. Moreover, geographic variability in its prevalence and clinical impact indicates that local environmental factors and healthcare practices influence its epidemiology. While typically opportunistic, *A. faecalis* can occasionally act as a primary pathogen in severe cases, such as endocarditis, meningitis, or septicemia [50].

A key challenge in managing *A. faecalis* infections lies in its ability to resist multiple classes of antibiotics. Several mechanisms contribute to its multidrug-resistant (MDR) phenotype. Producing β-lactamase enzymes, including carbapenemases, allows the bacterium to hydrolyze and inactivate β-lactam antibiotics, such as penicillins, cephalosporins, and carbapenems. Specific genes such as *bla_OXA-10_* and *bla_PER-1_* have been identified in *A. faecalis* strains, encoding β-lactamases with activity against a broad spectrum of β-lactams [53].

Efflux pumps further complicate treatment by actively expelling antibiotics, reducing their intracellular concentrations and effectiveness. The AcrAB-TolC efflux system, encoded by the *acr*AB genes, is a well-characterized multidrug efflux pump in Gram-negative bacteria, including *A. faecalis*. This system is critical in resistance to FQs, such as ciprofloxacin (CPX) and levofloxacin (LVX) [51]. Resistance to FQs is also mediated by mutations in the *gyr*A and *par*C genes, which encode the subunits of DNA gyrase and topoisomerase IV, respectively [23,51].

Another major contributor to resistance is its ability to form biofilms on medical devices and host tissues. Biofilms act as protective barriers, reducing the penetration of antibiotics and shielding bacteria from host immune responses. This biofilm-associated resistance is particularly problematic in infections involving prosthetic devices, catheters, and other medical implants [54]. In recent years, the emergence of extensively drug-resistant (XDR) strains of *A. faecalis* has shown resistance to almost all available antibiotics. In the 2019 study, susceptibility testing revealed that the best sensitivity rate was 66.7% for IMP, MEM, and CAZ, while CPX and P/T demonstrated sensitivity rates below 50% [51].

Treating *A. faecalis* infections requires a carefully tailored approach based on AST, as empirical antibiotic therapy is often ineffective due to the organism’s resistance profile. However, it is essential to remember that no specific breakpoints are available for EUCAST. In general, carbapenems, such as IMP and MEM, remain the first-line agents for treating serious infections caused by *A. faecalis*. However, the increasing prevalence of carbapenem-resistant strains has limited their utility. In cases involving XDR *A. faecalis*, tigecycline (TG) has emerged as a viable option, even demonstrating efficacy against strains resistant to carbapenems and other classes of antibiotics [51]. GNT and AMK may also be effective, mainly when combined with β-lactams, though their nephrotoxicity and ototoxicity require careful monitoring, especially in elderly patients. FQs, such as LVX, have been used for less severe infections, but their efficacy is increasingly undermined by resistance [53].

For biofilm-associated infections, adjunctive therapies targeting biofilm disruption are gaining attention. These include enzymatic agents that degrade the biofilm matrix and novel anti-biofilm compounds that enhance antibiotic penetration [54]. Emerging therapies, such as bacteriophages and antimicrobial peptides, are being explored for their potential to combat MDR and XDR pathogens, including *A. faecalis*. These approaches offer promise, particularly for infections refractory to conventional treatments.

Developing new antibiotic combinations, such as C/A, meropenem–vaborbactam (M/V), and FDC, represents another promising avenue [13,55,56]. While their efficacy against *A. faecalis* has not been extensively studied, these agents offer hope due to their activity against other resistant Gram-negative pathogens [13].

## 5. Burkholderia

Initially, *Burkholderia* species were classified under the Pseudomonadaceae family. In the early 1990s, they were included in the Burkholderiaceae family as *B. cepacia* [57,58]. The genus is divided into two large groups: *B. cepacia complex* (Bcc) and *B. pseudomallei complex* (Bpc) [59]. The two groups do not comprise all the species.

In the Bcc group, different species are included. These bacteria cause infections in humans, especially in immunocompromised and cystic fibrosis patients and in immunocompetent individuals [60,61]. The modifications occurring in the lung during this pathology are favorable for colonization by various pathogens, including Bcc, which is the most threatening for cystic fibrosis patients [62,63]. The most virulent species of the complex are *B. cenocepacia* and *B. multivorans* [59,64,65]. *B. cenocepacia*, due to its aggressive behavior and persistence in the lung airways, is considered a relative contraindication for lung transplants [66]. Despite the adequate treatment of the infections, the disease often results in chronic illness [66]. In some cases, Bcc species can develop the “cepacia” syndrome, characterized by necrotizing pneumonia and BSIs, usually affecting immunocompetent individuals [67,68]. Pathogens in the Bcc express many antibiotic-resistant mechanisms, it difficult to treat them treat correctly [59,69]. Also, the determination of antimicrobial susceptibility is a matter of debate. The CLSI offers the cut-off values of the main antibiotics employed in Bcc treatment. At the same time, the EUCAST has not set any cut-off values for any antibiotic under either method due to the few studies available and the absence of correlation between MICs obtained in vitro and the clinical result in vivo [70]. Moreover, Bcc expresses numerous intrinsic resistance patterns, such as resistance to aminopenicillin (excluded for piperacillin and P/T), cephalosporins (excluded CAZ and FEP), AZT ertapenem, CPX, chloramphenicol, AMGs, trimethoprim, Fosfomycin, and COL [27]. This complex situation is a threat to physicians in terms of the choice of the correct treatment. In vitro data and previous studies show that SXT, CAZ, MEM, and doripenem are the most effective antibiotics [48,71,72]. Minocycline (MIN) and doxycycline (DOXI) are considered good oral alternatives [73], but the choice is only supported by in vitro susceptibility. The new BL/BLI, including C/A and M/V, could be an alternative option, overwhelming the production of β-lactamases conferred by the *pen*A gene. Still, they succumb to efflux pump activity [74]. Eravacycline, a novel antibiotic of the TET family with broad-spectrum activity, is reported to have some activity against *B. cenocepacia,* but with MIC50/90 values of 8/32 g/L, which are relatively moderate/high [75]. The available studies suggest the use of combinations based on CAZ, MEM, doripenem, C/A, C/T, and FQs, coupled with FDC, for their in vitro activities [57,59,64,69,76,77]. In a summary of multinational surveillance studies, 94 Bpc species were collected and tested for FDC, with MIC90 values ranging from 0.03 to 1 g/L, suggesting good activity against Bpc pathogens [78]. Another study evaluated the susceptibility of FDC of the various non-fermenter species, including 7 isolates of Bpc with all strains inhibited by FDC with MIC < 25 g/L [79]. Delafloxacin (DELA), a fourth-generation quinolone, has been evaluated in vitro against Bcc pathogens. In detail, 57 isolates of Bcc were tested: MIC50 values were 0.25 g/L for *B. cepacia* and *B. multivorans* and 2 g/L for *B. cenocepacia*. The authors concluded by showing the potential activity of DELA [80]. Other therapeutic strategies include a combination of inhalation drugs with intravenous administration, in particular, inhaled tobramycin with FDC [64] or prolonged ATZ [81,82]. However, results are scarce. Furthermore, new therapies, such as bacteriophages [83] and large-molecule polycationic glycopolymers, are under evaluation for clinical practice, with the latter restoring antibiotic activity [84]. Finally, new compounds based on auranofin analogs have shown promising results in eradicating persistent infection, thus enabling newer treatment options, alone or combined with antibiotics [85].

The Bpc group includes *B. mallei* (the causative agent of glanders) [86], *B. pseudomallei* (the causative agent of Melioidosis) [87], *B. humpydoensis*, and *B. thailandensis* [59,88]. *B. mallei* and *B. pseudomallei* are considered biological weapons [86]. Although both infections can lead to severe disease with high mortality rates if not promptly treated, *B. pseudomallei* is far more common in humans than *B. mallei* and expresses more sophisticated mechanisms of antimicrobial resistance [59,88,89,90]. *B. pseudomallei* is considered a tropical pathogen. It is usually encountered in Thailand, but is likely to be widespread in Southeast Asia, the Indian subcontinent, Sri Lanka, China, and Papua Nuova Guinea [91,92,93]. Clinical manifestations of *B. pseudomallei* are varied, ranging from cutaneous disease to sepsis syndrome with necrotizing pneumonia [89]. Also, the disease can be divided into four types: (i) an acute type, (ii) a subacute type, (iii) a chronic type, and (iv)a latent or asymptomatic type [89,94]. In the acute type, there are two stages of therapy: the intensive phase and the eradication phase [89,94]. Notably, although the Bpc pathogens share a lot of genetic homologies, *B. pseudomallei* has more complex gene expression than *B. mallei* and *B. thailandensis*, ultimately leading to much more pronounced antimicrobial resistance [59,95]. *B. pseudomallei* is intrinsically resistant to anti-bactericidal penicillins [except for amoxicillin–clavulanic acid (A/C)], first-, second-, and third-generation cephalosporins (except for CAZ), AMGs, and rifamycins [91,94,96,97]. For Bpc, EUCAST has different interpretation breakpoints for the principal antibiotic used in case of infections (i.e., amoxicillin–clavulanic acid, CAZ, IMP, MEM, DOXI, TET and SXT) [47]. The intensive phase is based on intravenous treatment with CAZ, MEM, or IMP [89,90,94,98,99], with a minimum duration of therapy of 2 weeks. This lasts up to 4–6 weeks if metastatic infection coexists [94,100]. The previously cited antibiotics can be coupled with SXT if deep-seated infections exist. The eradication phase begins when the patient is hemodynamically stable, the C-reactive protein (CRP) levels have fallen, and any abscesses or deep-seated infections have significantly improved or been resolved [90,94,99]. This phase lasts 3 to 6 months and consists of SXT, alone or combined with DOXI or A/C [89,94]. Treatment for *B. mallei* is similar to that for *B. pseudomallei*, consisting of intravenous therapy with CAZ, MEM, or IMP [99,101] and oral SXT, A/C, or DOXI [99,101,102]. Among new antimicrobial therapies, Burnard et al. tested 246 *B. mallei* clinical isolates for FDC, showing MICs ranging from 0.03 to 16 g/L [103]. Tackling the antimicrobial resistance patterns of Bpc, new therapeutic strategies have been studied. Antibiotic treatments with C/T, finafloxacin, rifampicin, auranofin, doripenem, ertapenem, TG, moxifloxacin (MOXI), and MMV688271 (a new antifungal agent) have been tested for *B. pseudomallei*, showing in vitro susceptibility [104]. Alongside these drugs, potentiation compounds have been evaluated, like silver nanoparticles (AgNPs) and NanoClusters [104]. Also, the use of antibody therapy, antimicrobial peptides, and phage therapy has been proposed [104]. For *B. mallei*, antibiotic potentiation, with a whole-killed vaccine coupled with MOXI, SXT, or azithromycin, has been suggested for treatment [104]. Resistant mechanisms are shown in Table 2.

*B. gladioli* are commonly found in water and soil and can serve as nosocomial pathogens. While infections are rare, occasional outbreaks have been documented, and the development of multidrug resistance remains a potential issue [19]. *B. gladioli* has been documented as a cause of disease in patients with cystic fibrosis, chronic granulomatous disease and other immunocompromising conditions [19,110]. No specific breakpoints are available, and this could represent particular challenges due to its potential for multidrug resistance. AST should be guided by susceptibility testing. Standard treatment options include SXT, MEM, IMP, and FQ. In severe cases, combination therapy may be necessary, and surgical intervention might be required for localized infections such as abscesses. For patients with cystic fibrosis or other immunocompromising conditions, prolonged treatment and careful monitoring are essential to prevent recurrence [111].

## 6. Elizabethkingia

The bacteria belonging to the *Elizabethkingia* genus are aerobic, non-fermenting, non-motile, catalase-positive, oxidase-positive, indole-positive, and Gram-negative bacilli belonging to the family Flavobacteriaceae. They were first described by Centers for Disease Control and Prevention (CDC) microbiologist Elizabeth O. King in 1959 [112,113]. The genus was initially classified as Flavobacterium. It was then reclassified as Chryseobacterium in 1994, and received its current taxonomic designation in 2005 [114]. It has an environmental distribution (e.g., water, soil), but can also colonize hospital settings [113].

In recent years, there has been an increase in reported outbreaks of *Elizabethkingia* infections, likely due to improved identification methods (e.g., MALDI-TOF) and a rise in the number of immunocompromised individuals. Countries reporting numerous cases include Saudi Arabia and India; a link may exist between warmer climates and mosquitos [115,116].

Infections caused by *Elizabethkingia* spp. have been reported worldwide, with documented outbreaks in North America, Europe, Asia, and Africa. The outbreaks often occur in hospital settings, especially in intensive care units (ICUs) and neonatal wards. Currently, this genus is known to comprise eight species, which include *E. meningoseptica*, *E. anophelis, E. miricola*, *E. bruuniana*, *E. ursingii*, *E. argenteiflava*, *E. umeracha*, and *E. occulta* [114,117,118]. However, *E. meningoseptica*, *E. anophelis*, and *E. miricola* are the most common species. Historically, *E. meningoseptica* was the most commonly isolated species in the genus. However, *E. anopheles* has emerged as the predominant pathogen, accounting for 59–99% of clinical isolates, while *E. meningoseptica* constitutes only about 1–21% (Lin et al., 2019). This apparent shift may be due to improved identification techniques, which mainly use MALDI-TOF [113].

**Figure 5:**
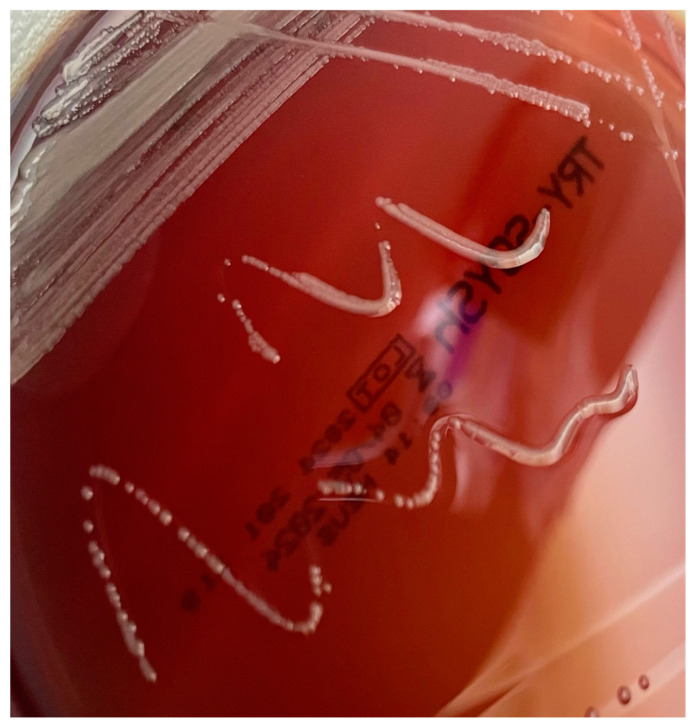
*Elizabethkingia anophelis* colonies on TSA appear large, mucoid, and translucent, with an irregular and wavy growth pattern. They have a glistening, moist texture without visible hemolysis.

*Elizabethkingia* can cause various infections, including meningitis, bloodstream infections (BSIs), pneumonia, urinary tract infections (UTIs), and skin and soft tissue infections (ABSSIs). Infections are particularly problematic in newborns and immunocompromised patients, and are recognized in particular for causing neonatal sepsis and meningitis, particularly in premature infants [114]. A large case series in children, reported in a review by Dziuban et al., demonstrated that 73.9% of cases presented with meningitis, 23.7% with sepsis, 6.7% with pneumonia, and 2.5% with gastroenteritis/diarrhea [114]. *Elizabethkingia* infections are implicated in outbreaks of severe infections in about one-third of cases, with mortality rates between 24% and 60% [113,114,119,120,121]. Typically, these outbreaks are linked to many sources, including contaminated saline solutions, respiratory equipment, and skin drains [122]. Figure 5 shows *E. anophelis* growing in the Petri dish.

*Elizabethkingia* spp. is known for its resistance to many commonly used antibiotics, making the treatment of infections difficult. It constitutively produces β-lactamases and is naturally resistant to most β-lactam drugs, including carbapenems and AZT, except for piperacillin and P/T. Three β-lactamases were identified in *Elizabethkingia*: one D-class serine (CME) and two wide-spectrum MBLs with carbapenemase activity, namely, BlaB, and GOB [123]. No breakpoints are available for EUCAST, and for this reason most studies use breakpoints, which are interpreted according to CLSI guidelines. These showed that the most effective agents for in vitro susceptibility were MIN (100%), LVX (65–80%), and SXT (63–90%). Sensitivity to carbapenems was less than 2% and sensitivity to cephalosporins and AMG was also low [123,124]. Additionally, other researchers reported high sensitivity to rifampin (94%) [125]. Both in vitro and in vivo data on FDC for *Elizabethkingia* infections are scant; however, a case series of 22 CPX-non-susceptible strains tested for FDC revealed high MICs (>32 mg/L) [126]. Notably, *Elizabethkingia* can produce biofilms, with over one-third of strains being strong biofilm formers [125]. This is especially significant when the respiratory tract is affected, such as ventilator-associated pneumonia in patients with bronchiectasis [127].

## 7. Moraxella

*Moraxella* is a genus of Gram-negative, aerobic, oxidase-positive bacteria, with *M. catarrhalis* being the most clinically significant species [128]. *M. catarrhalis* commonly colonizes the human upper respiratory tract and is associated with various infections [129,130]; commonly, it causes otitis media and sinusitis, especially in children, and lower respiratory tract infections, especially in people with chronic obstructive pulmonary disease [131,132,133]. Invasive infections are less common but cases have been reported of BSIs, endocarditis, septic arthritis, and meningitis, especially in immunocompromised patients [134]. Other less common species are *M. lacunata*, *M. osloensis*, *M. nonliquefaciens*, and *M. bovis* [134]. These species rarely cause infection in immunocompetent individuals. *M. osloensis*, typically a commensal organism, has been associated with BSIs and septic arthritis in immunocompetent individuals [135]. Similarly, *M. lacunata*, *M. nonliquefaciens*, and *M. bovis* are generally considered part of the normal human flora but can occasionally cause infections, particularly in individuals with cancer or compromised immune systems [136,137,138,139].

**Figure 6:**
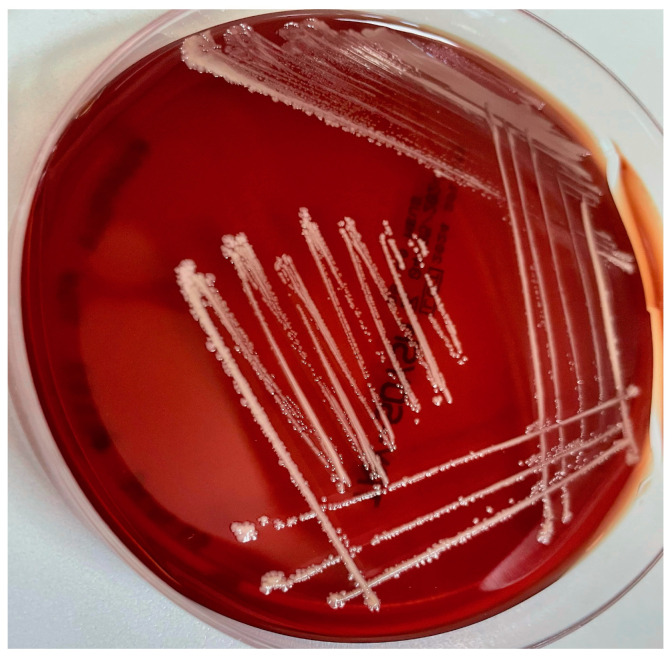
*Moraxella catarrhalis* colonies on TSA appear pale and whitish. The colonies are medium-sized, irregular, and slightly rough in texture, with a matte or granular surface and without visible hemolysis.

*M. catarrhalis* is also known for producing β-lactamase enzymes, contributing to its antibiotic resistance profile. In particular, studies have reported that β-lactamase production rates in *M. catarrhalis* isolates range from 80% to 99% [140,141,142]. These enzymes, primarily BRO-1 and BRO-2, hydrolyze the β-lactam ring of susceptible antibiotics, rendering them ineffective. BRO-1 is more prevalent and typically associated with higher MICs for penicillin varieties compared to BRO-2 [143]. These enzymes are synthesized in the cytoplasm and transported to the periplasmic space via the twin-arginine translocation (Tat) pathway [144]. However, β-lactamase inhibitors such as clavulanic acid can inactivate these β-lactamases [145,146]. Additionally, *M. catarrhalis* releases outer membrane vesicles containing β-lactamases, which can inactivate β-lactam antibiotics in the extracellular environment, potentially protecting neighboring bacteria and complicating polymicrobial infections [147,148]. The high prevalence of β-lactamase production in *M. catarrhalis* necessitates careful selection of antibiotic therapy. Despite that, A/C remains the treatment of choice, as other molecules can be associated with other resistant mechanisms. Also, resistance to SXT, macrolides, and TET has been reported, underscoring the importance of local susceptibility patterns in guiding effective treatment [149,150,151,152]. Fortunately, the interpretation of AST is permitted by the presence of breakpoints for EUCAST and CLSI [47,48]. Figure 6 shows evidence of *M. catarrhalis* colonies on TSA.

Regarding new antibiotic treatments, limited data are available on the efficacy of agents such as FDC, M/V, C/A and C/T against *M. catarrhalis*. These antibiotics have shown promise against various MDR Gram-negative bacteria; however, their specific activity against *M. catarrhalis* has not been studied extensively.

## 8. Other Rare Non-Fermenting Gram-Negative Bacteria

Many other NFGNB have a clinical impact on human infections [15], further complicating treatment decisions due to the lack of specific breakpoints and standardized therapeutic approaches.

*Ochrobactrum* spp. are non-enteric, Gram-negative organisms closely phylogenetically related to the genus *Brucella*. The taxonomic classification is still problematic for *Ochrobactrum* spp., with publication by bacterial taxonomists who included *Ochrobactrum* within the genus *Brucella* [153]. Even though they are considered pathogens with low virulence, clinical reports have increasingly been described in the literature [154,155]. Most cases were related to hospital-acquired infections, immunocompromised hosts, or patients with tumors [155,156]. Species isolates from human samples are *O. anthropic*, *O. intermedium*, *O. oryzae*, *O. pseudogrignonense*, *O. pseudintermedium,* and *O. tritici* [155]. Treatment is often complex due to the emerging problem of antibiotic resistance. In particular, many strains show resistance to penicillins, cephalosporins and, in some cases, carbapenems [157]. Resistance to β-lactam antibiotics is associated with a chromosomal gene (*bla_och_*) similar to the Ambler-class C β-lactamase gene. This gene encodes an AmpC-like enzyme called OCH [158]. Leading carbapenem resistance, a plasmid-borne *bla_oxa-181_* gene has been found in some *O. intermedium* strains [159]. Although this genus could show resistant mechanisms, *Ochrobactrum* maintains susceptibility to CPX and SXT, suggesting that the combination of these two drugs may be helpful for the empirical treatment of *Ochrobactrum* infections [157]. Moreover, in most cases described in the literature, AMG, FQ, carbapenem, and SXT were used alone or as a part of the combination treatment [155].

*Bergeyella* spp. are uncommonly identified as a cause of human disease, often presenting as ABSSIs, BSIs, and infective endocarditis [160]. *B. zoohelcum* is the principal human pathogen, and it has been isolated from wound infections following animal bites but also in severe human infections, such as BSIs, endocarditis, and meningitis [160,161]. *Bergeyella* is usually susceptible to most antimicrobial agents, such as β-lactams and FQs. Successful treatment with ampicillin–sulbactam, A/C, cefazolin with GNT, cefuroxime, and CPX has been reported [160,162,163].

*Weeksella virosa* is rarely and uncommonly implicated in infections in humans. It is clinically associated with BSIs, peritonitis, pneumonia, and UTIs in immunocompromised patients and, in many cases, related to nosocomial infections [164,165]. Typical empirical treatment should include piperacillin, AZT, or carbapenems; instead, SXT, CPX and AMG should not be used unless AST is available [164,166].

*Chryseobacterium* spp. are ubiquitous in soil and water and have also been recovered from foods and the hospital environment [167]. *C. indologenes* is the variant most frequently isolated from human specimens [168]. Most reported cases are nosocomial, and are often associated with immunosuppression or indwelling catheters. *Chryseobacterium* spp. has been reported in the cases of BSIs, peritonitis, pneumonia, empyema, pyelonephritis, cystitis, meningitis, and central venous catheter (CVC)-related infections [169]. *C. indologenes* is intrinsically resistant to penicillin (excluding piperacillin and P/T), AZT, third-generation cephalosporins (excluding FEP), carbapenem, and AMG [27]. Moreover, it is uniformly resistant to erythromycin, clindamycin, vancomycin, and teicoplanin [15]. The most potent agents reported against *Chryseobacterium* spp. are quinolones (garenoxacin, gatifloxacin, and LVX, each with 98.0% susceptibility), SXT (>95% susceptibility), and rifampin (85.7% susceptibility) [170].

The family Comamonadaceae includes the genera *Comamonas*, *Delftia* and *Acidovorax*. *Comamonas* includes *C. aquatica*, *C. kerstersii*, *C. terrigena,* and *C. testosteronei*. The genus *Delftia* consists of *D. acidovorans*, formerly designated *C. acidovorans*. Finally, *Acidovorax* includes *A. facilis*, *A. delafieldii,* and *A. temperans* [15]. Rare cases of CVC-related BSIs (*C. testosteroni*, *D. acidovorans*, *Acidovorax* spp.), meningitis (*C. testosteroni*), endocarditis (*C. testosteroni*, *D. acidovorans*), conjunctivitis (*C. testosteroni*), and otitis media (*D. acidovorans*) have been reported [15]. The antibiotic treatment of *Comamonas* infections can be particularly challenging. Most clinical isolates of *Comamonas* are susceptible to various antibiotics, including P/T, cephalosporins, carbapenems, FQ, SXT, and AMG antibiotics [171]. However, in several cases, *C. testosteroni* was reportedly resistant to CPX, GNT, and CAZ [172]. Resistance to β-lactams class antimicrobials can occur due to the presence of several genes encoding Class A β-lactamases, oxacillinases (i.e., *bla_OXA-1_*), and carbapenemases (i.e., *bla_IMP-8_* and *bla_GES-5_*) [171]. *D. acidovorans* usually show susceptibility to CPX (90.7%), CAZ (94.4%), P/T (94.0%) and carbapenems (MEM susceptibility 94.6%, IMP susceptibility 94.2%) [173,174]. AMG should not be used because of the high risk of resistance [173]. *Acidovorax* infections are so rare that no general treatment recommendations can be made; in some cases, patients are treated with FQ or P/T [175].

The genus *Oligella* includes *O. urethralis* (derived from *M. urethralis* and CDC group M-4) and *O. ureolytica* (derived from CDC group IVe). Both species have been implicated in BSIs, arthritis, and genitourinary infections [19,176]. *O. urethralis* is intrinsically susceptible to penicillins, cephalosporins, and carbapenems. In contrast, *O. ureolytica* can display decreased susceptibility to ampicillin or amoxicillin, suggesting chromosomal encoding of the penicillinase gene. FQ, AMG, and SXT show inconsistent activity in vitro against *O. ureolytica* [176].

*Pandoraea* spp. are usually isolated from soil, water, plants, fruits and vegetables. The most frequent infections are pneumonia (in many cases related to cystic fibrosis) and BSIs. Upper respiratory infections, osteomyelitis, pancreatitis, and endocarditis are rare [177]. *Pandoraea* spp. is considered an MDR pathogen, and most species resist β-lactams and AMG [177]. A possible explanation for MDR is the germ’s production of certain enzymes, carbapenemases (i.e., OXA-62), and efflux pumps [178]. In cases of systemic infection, aggressive antimicrobial treatment is mandatory to reduce the risk of fatal outcomes. It could include carbapenems, especially IMP, or cephalosporins with SXT, followed by AMG and FQ [177].

The genus *Psyrobacter* is an extremely rare human pathogen. Some cases are reported in meningitis, wound infections, and BSIs [179].

The genus *Ralstonia* is emerging as an opportunistic human nosocomial pathogen in immunocompromised patients, especially in persons with cystic fibrosis [180]. This genus comprises three species of human interest: *R. insidiosa*, *R. mannitolilytica*, and *R. pickettii* [180]. *Ralstonia* spp. are generally MDR [181]. SXT and FQ antibiotics are considered the best treatment options [180,181]. However, TG also shows good in vitro activity against *Ralstonia* isolates [180]. Different resistant mechanisms were associated with this bacteria, including efflux pumps and β-lactamases (OXA-22-like and OXA-60-like subfamily) [180].

*Rhizobium* spp. are phytopathogenic organisms in water, soil, and environmental plants. They are rarely associated with human infections. However, some cases are associated with *R. radiobacter* infections [182,183]. *Rhizobium* spp. are generally susceptible to cephalosporins (second- and third-generation), ticarcillin, IMP, TET, COL, SXT, and FQs [19].

*Shewanella* spp. is a genus of NFGNB that is rarely an opportunistic human pathogen [184]. A variety of factors can influence the infection. Firstly, *Shewanella* spp. are distributed in water environments. Recreational or occupational exposure, seafood ingestion, puncture wounds caused by marine organisms, or the direct exposure of a wound to aquatic environments can increase the risk of infection [185]. Secondly, *Shewanella* can be found in patients with immunocompromised states, including malignancies, severe heart failure, hepato-renal failure, and neutropenia [185]. Several *Shewanella* spp. have recently emerged as worldwide pathogens, including *S. algae, S. putrefaciens*, and *S. xiamenensis* [186,187,188]. *Shewanella* spp. are generally resistant to penicillins but susceptible to third-generation cephalosporins, IMP, CPX, AMGs, SXT, and TETs [19].

Bacteria belonging to the *Sphingobacterium* are ubiquitous but rarely involved in human infections [189]. The few reported diseases resulting from *Sphingobacterium* usually occurred in severely comorbid patients and immunocompromised hosts [190].

*Sphingobacterium* spp. in vitro are usually intrinsically resistant to many commonly used antibiotics and can grow in antiseptics and disinfectants [191]. *S. multivorum* can produce extended-spectrum β-lactamase (ESBL) and MBL, resulting in resistance to penicillins, cephalosporins and carbapenems [192]. In addition, *Sphingobacterium* spp. show resistance to AMG and polymyxin B and are susceptible to FQ, TET, and SXT [190].

In Table 3, we summarize the possible therapeutic options for different NFGNB.

## 9. Discussion

Rare or unusual NFGNB are emerging as increasingly important pathogens in community and healthcare settings, particularly among immunocompromised individuals and patients with underlying conditions. Despite their diverse taxonomy, these organisms share a common challenge: their intrinsic and acquired resistance mechanisms, which limit effective antibiotic options and complicate treatment strategies. The epidemiology of these rare NFGNB varies significantly, with some species like *Burkholderia* and *Achromobacter* being more frequently associated with chronic infections, such as in cystic fibrosis patients [43,109]. In contrast, others, like *Elizabethkingia* and *Moraxella*, are more often linked to opportunistic infections in critically ill individuals [127,140]. Their ability to persist in hospital environments and form biofilms further contributes to outbreaks and increased morbidity.

Antibiotic resistance among these pathogens is a significant concern. Many exhibit resistance to β-lactams, AMG, and FQ due to various mechanisms, including efflux pumps, β-lactamase production, and target site modifications [142]. Treatment options are often limited and require tailored approaches based on susceptibility testing. Novel BL/BLI combinations or FDC have shown promise in select cases. Combination therapy may be necessary in severe infections, especially in multidrug-resistant strains. Given the limited clinical data and the absence of standardized treatment guidelines for many of these organisms, further research is needed to establish optimal therapeutic regimens. Developing new antimicrobial agents, improved stewardship, and rapid diagnostic techniques will be critical in managing infections caused by these rare but increasingly relevant pathogens.

## 10. Materials and Methods

A comprehensive literature search was conducted to identify relevant studies concerning NFGNB infections, particularly on rare bacteria. The search strategy was implemented using online databases [PubMed (USA)/MEDLINE (USA)/Google Scholar (Google, Mountain View, CA, USA) ] and books written by experts in microbiology and infectious diseases. The search was not restricted by language, region, study type, or publication date and covered articles up to the cutoff date of January 2025. Conference abstracts or unpublished studies are also included. The following keywords and MeSH were used: “*Achromobacter* AND human infections”, “*Achromobacter* AND treatment”, “*Achromobacter* AND resistance mechanism”, “*Alcaligenes* AND human infections”, “*Alcaligenes* AND treatment”, “*Alcaligenes* AND resistance mechanism”, “*Burkholderia* AND human infections”, “*Burkholderia* AND treatment”, “*Burkholderia* AND resistance mechanism”, “*Elizabethkingia* AND human infections”, “*Elizabethkingia* AND treatment”, “*Elizabethkingia* AND resistance mechanism”, “*Moraxella* AND human infections”, “*Moraxella* AND treatment”, “*Moraxella* AND resistance mechanism”, “*Ochrobactrum* AND human infections”, “*Ochrobactrum* AND treatment”, “*Ochrobactrum* AND resistance mechanism”, “*Bergeyella* AND human infections”, “*Bergeyella* AND treatment”, “*Bergeyella* AND resistance mechanism”, “*Weeksella* AND human infections”, “*Weeksella* AND treatment”, “*Weeksella* AND resistance mechanism”, “*Chryseobacterium* AND human infections”, “*Chryseobacterium* AND treatment”, “*Chryseobacterium* AND resistance mechanism”, “*Comamonas* AND human infections”, “*Comamonas* AND treatment”, “*Comamonas* AND resistance mechanism”, “*Delftia* AND human infections”, “*Delftia* AND treatment”, “*Delftia* AND resistance mechanism”, “*Acidovorax* AND human infections”, “*Acidovorax* AND treatment”, “*Acidovorax* AND resistance mechanism”, “*Oligella* AND human infections”, “*Oligella* AND treatment”, “*Oligella* AND resistance mechanism”, “*Pandoraea* AND human infections”, “*Pandoraea* AND treatment”, “*Pandoraea* AND resistance mechanism”, “*Psyrobacter* AND human infections”, “*Psyrobacter* AND treatment”, “*Psyrobacter* AND resistance mechanism”, “*Ralstonia* AND human infections”, “*Ralstonia* AND treatment”, “*Ralstonia* AND resistance mechanism”, “*Rhizobium* AND human infections”, “*Rhizobium* AND treatment”, “*Rhizobium* AND resistance mechanism”, “*Shewanella* AND human infections”, “*Shewanella* AND treatment”, “*Shewanella* AND resistance mechanism”, “*Sphingobacterium* AND human infections”, “*Sphingobacterium* AND treatment”, “*Sphingobacterium* AND resistance mechanism”, “Rare Non-fermenting Gram-negative AND human infections”, “Non-fermenter bacteria AND human infections”, “Unusual AND Non-fermenting Gram-negative”, “Rare AND Non-fermenting Gram-negative”, “Unusual AND Non-fermenter bacteria”, “Rare AND Non-fermenter bacteria”. We screened the articles by title, abstract and full text. After an initial screening of titles and abstracts of the published articles, the reviewers evaluated the full articles to assess the eligibility for each study’s inclusion in this narrative review. A study was included to determine if it was likely to provide valid and valuable information according to their view’s objective.

## 11. Conclusions

Rare NFGNB pose an increasing challenge, particularly in immunocompromised patients and critically ill settings. Their intrinsic and acquired resistance limits treatment options, making susceptibility-guided therapy essential. In many cases, treatments are not guided by solid evidence-based literature. Enhancing diagnostic strategies, developing new antibiotics, and implementing research programs on this rare pathogenic entity will be crucial to optimizing infection management and combating resistance spread.

## Figures and Tables

**Table 1:** *Achromobacter* spp. common resistant mechanisms.

Type of Resistant Mechanism	Antibiotics Affected	Reference
**Multidrug Efflux Pumps**		[32,41]
AxyABM	Cephalosporins (except cefuroxime and FEP), AZT and chloramphenicol	
AxyXY-OprZ	AMG, TET, TG, FQ, FEP, carbapenems	
**β-Lactamases**		[32]
OXA-114-like	Piperacillin, ticarcillin, benzylpenicillin, cephalothin	
ESBL and AmpC	All β-lactams except carbapenems	
Metallo-β-lactamases	All β-lactams except AZT	
**Other mechanisms**		[32,42]
aac(6′)Ib-cr, qnrA, oqxA, oqxB	FQ, AMG	
*gyrA*, *ParC*	FQ	
Biofilm	β-lactams and AMG	

AMG = aminoglicoside, AZT = aztreonam, FEP = cefepime, FQ = fluoroquinolone, TET = tetracycline, TG = tigecycline.

**Table 2:** *Burkholderia* spp. common resistant mechanisms.

Type of Resistant Mechanism	Antibiotics Affected	Reference
Class A β-lactamases (gene *penA*, *penB* and *penR*)	Mutations lead to CAZ resistance, IMP, A/C	[59]
The efflux pump system of the resistance nodulation cell division	Intrinsic resistance to penicillin, first and second-generation cephalosporins, gentamycin, tobramycin, streptomycin, polymyxin	[91,96,105]
Reduced outer membrane permeability/modified LPS structure	Polymixin	[59]
Alteration in drug targets	Mutations affecting topoisomerases type II enzymes, DNA gyrase, and topoisomerases type IV leads to FQ resistance. Mutations in the dihydrofolate reductase led to SXT resistance	[59,106,107]
Biofilm	Unlike planktonic organisms, *B. pseudomallei* biofilm production is associated with resistance to multiple antimicrobials, including CAZ, IMP, SXT	[108,109]

A/C = amoxicillin–clavulanic acid, CAZ = ceftazidime, DNA = deoxyribonucleic acid, FQ = fluoroquinolone, IMP = imipenem, LPS = lipopolysaccharide, SXT = trimethoprim–sulfamethoxazole.

**Table 3:** Empirical antibiotic treatment suggested for NFGNB infections.

Microorganism	Mild to Moderate Disease	Sever Disease *	References
*Achromobacter* *xylosoxidans*	No prior antibiotic exposure Ceftazidime Trimethoprim/sulfamethoxazole Ciprofloxacin Prior antibiotic exposure Meropenem or imipenem cilas-tatin Cefiderocol Eravacycline	No prior antibiotic exposure Ceftazidime + trimethoprim/sulfamethoxazole Meropenem or Imipenem cilastatin + Trimethoprim/sulfamethoxazole Prior antibiotic exposure Cefiderocol + trimethoprim/sulfamethoxazole You can consider also ciprofloxacin as a part of the treatment	[32,49]
*Alcaligenes* *faecalis*	Meropenem or imipenem cilastatin Tigecycline Fluoroquinolone	No prior antibiotic exposure Meropenem or imipenem cilastatin prior antibiotic exposure Meropenem or imipenem cilastatin + tigecycline Meropenem or imipenem cilastatin + Aminoglycoside Meropenem or imipenem cilastatina + Fluoroquinolones	[51,53]
*Burkholderia * *cepacia* complex	Trimethoprim/sulfamethoxazole Fluoroquinolone Ceftazidime Minocycline or doxycycline	No prior antibiotic exposure Trimethoprim/sulfamethoxazole Ceftazidime Prior antibiotic exposure Ceftazidime + trimethoprim/sulfamethoxazole Meropenem or doripenem + trimethoprim/sulfamethoxazole Alternatives Cefiderocol (need prior testing) + one or more antibiotics previously cited Ceftazidime/avibactam (need prior testing) ± one or more antibiotics previously cited Meropenem/vaborbactam (need prior testing) ± one or more antibiotics previously cited	[48,71,72,73,74,75]
*Burkholderia pseudomallei* complex	IV initial therapy Ceftazidime Meropenem Eradication therapy Trimethoprim/sulfamethoxazole		[89,94]
*Burkholderia* *gladioli*	Trimethoprim/sulfamethoxazole Meropenem or imipenem cilastatin Fluoroquinolone	No prior antibiotic exposure Trimethoprim/sulfamethoxazole + fluoroquinolone Prior antibiotic exposure Meropenem or imipenem cilastatin + trimethoprim/sulfamethoxazole Meropenem or imipenem cilastatin + fluoroquinolone	[111]
*Elizabethkingia* *meningoseptica*	Minocycline Levofloxacin Trimethoprim/sulfamethoxazole	No prior antibiotic exposure Minocycline + Levofloxaicin Minocycline + trimethoprim/sulfamethoxazole Trimethoprim/sulfamethoxazole + Levofloxacin Prior antibiotic exposure Same combinations, eventually with rifampin or cefiderocol	[124,125,126]
*Moraxella* *catharallis*	Amoxicillin/clavulanic acid alternatives: Azitromycin Trimethoprim/sulfamethoxazole		[149,150,151,152]
*Ochrobactrum* spp.	Ciprofloxacina + trimethoprim/sulfamethoxazole		[155,157]
*Bergeyella* spp.	Ampicillina/sulbactam or Amoxicillina/clavulanic acid Cefazolin + gentamycin Cefuroxime Ciprofloxacin		[160,162,163]
*Weeksella virosa*	Piperacillin Aztreonam Carbapenem		[164,166]
*Chryseobacterium* spp.	Levofloxacin Trimethoprim/sulfamethoxazole Eventually, consider rifampin as a part of the treatment		[170]
*Comamonas* spp.	Piperacillin/tazobactam Carbapenems Trimethoprim/sulfamethoxazole Consider combination therapy based on the risk of resistant strains		[171]
*Delftia* *acidovorans*	Ceftazidime Piperacillin/tazobactam Meropenem or imipenem cilastatin Ciprofloxacin		[173,174]
*Acidovorax* spp.	Piperacillin/tazobactam Fluoroquinolones		[175]
*Oligella* spp.	Cephalosporins Carbapenems		[176]
*Pandoraea* spp.	Imipenem cilastatin + trimethoprim/sulfamethoxazole		[177]
*Ralstonia* spp.	Fluoroquinolone + trimethoprim/sulfamethoxazole Eventually, consider tigecycline as a part of the treatment		[180,181]
*Rhizobium* spp.	Cefalosporins Ticarcillin Imipenem cilastatin Tetracyclines Colistin Fluoroquinolones Trimethoprim/sulfamethoxazole Consider combination therapy based on the risk of resistant strain		[19]
*Shewanella* spp.	Cefalosporins Imipenem cilastatin Tetracyclines Aminoglycosides Fluoroquinolone Trimethoprim/sulfamethoxazole Consider combination therapy based on the risk of resistant strain		[19]
*Sphingobacterium* spp.	Fluoroquinolones Tetracyclines Trimethoprim/sulfamethoxazole Consider combination therapy based or the risk of resistant strain		[190,192]

* The literature does not provide studies with sufficient scientific robustness to recommend monotherapy, even in cases of antibiotic susceptibility, for severe infections. Combination therapy is suggested to overcome pathogen resistance. Moreover, true clinical breakpoints are currently lacking for the majority of the antibiotics tested; therefore, in vitro susceptibility data may not have a reliable clinical correlation.

## Data Availability

Not applicable.

## Abbreviations

The following abbreviations are used in this manuscript:

ABSSI	acute bacterial skin and skin structure infection
A/C	amoxicillin–clavulanic acid
AMG	aminoglycoside
AMK	amikacin
AST	antimicrobial susceptibility testing
AZT	aztreonam
Bcc	*B. cepacia complex*
BL/BLI	β-lactam/β-lactamase inhibitor
Bpc	*B. pseudomallei complex*
BSI	bloodstream infection
CAZ	ceftazidime
CDC	Centers for Disease Control and Prevention
C/A	ceftazidime–avibactam
CLSI	Clinical and Laboratory Standards Institute
CRP	C-reactive protein
C/T	ceftolozane–tazobactam
DELA	delafloxacin
DOXI	doxycycline
ESBL	extended-spectrum β-lactamase
EUCAST	European Committee on Antimicrobial Susceptibility Testing
FEP	cefepime
FDC	cefiderocol
FQ	fluoroquinolone
GNT	gentamicin
ICU	intensive care unit
IMP	imipenem
LPS	lipopolysaccharide
MALDI-TOF	matrix-assisted laser desorption/ionization time of flight
MBL	metallo-β-lactamase
MDR	multidrug-resistant
MEM	meropenem
M/V	meropenem–vaborbactam
MIC	minimum inhibitory concentration
MIN	Minocycline
MOXI	Moxifloxacin
NFGNB	non-fermenting Gram-negative bacteria
OXA	oxacillinase
PCR	polymerase chain reaction
P/T	piperacillin–tazobactam
TET	tetracycline
TG	tigecycline
UTI	urinary tract infection
XDR	extensively drug-resistant

## References

[1] Enoch D.A., Birkett C.I., Ludlam H.A. (2007). Non-Fermentative Gram-Negative Bacteria. Int. J. Antimicrob. Agents.

[2] Di Pilato V., Willison E., Marchese A. (2023). The Microbiology and Pathogenesis of Nonfermenting Gram-Negative Infections. Curr. Opin. Infect. Dis..

[3] Behzadi P., Baráth Z., Gajdács M. (2021). It’s Not Easy Being Green: A Narrative Review on the Microbiology, Virulence and Therapeutic Prospects of Multidrug-Resistant Pseudomonas Aeruginosa. Antibiotics.

[4] Giovagnorio F., De Vito A., Madeddu G., Parisi S.G., Geremia N. (2023). Resistance in Pseudomonas Aeruginosa: A Narrative Review of Antibiogram Interpretation and Emerging Treatments. Antibiotics.

[5] Casale R., Boattini M., Comini S., Bastos P., Corcione S., De Rosa F.G., Bianco G., Costa C. (2024). Clinical and Microbiological Features of Positive Blood Culture Episodes Caused by Non-Fermenting Gram-Negative Bacilli Other than Pseudomonas and Acinetobacter Species (2020–2023). Infection.

[6] Yadav S.K., Bhujel R., Mishra S.K., Sharma S., Sherchand J.B. (2020). Emergence of Multidrug-Resistant Non-Fermentative Gram Negative Bacterial Infection in Hospitalized Patients in a Tertiary Care Center of Nepal. BMC Res. Notes.

[7] Mojica M.F., Humphries R., Lipuma J.J., Mathers A.J., Rao G.G., Shelburne S.A., Fouts D.E., Van Duin D., Bonomo R.A. (2022). Clinical Challenges Treating Stenotrophomonas Maltophilia Infections: An Update. JAC Antimicrob. Resist..

[8] Rattanaumpawan P., Ussavasodhi P., Kiratisin P., Aswapokee N. (2013). Epidemiology of Bacteremia Caused by Uncommon Non-Fermentative Gram-Negative Bacteria. BMC Infect. Dis..

[9] Rivera-Villegas H.O., Martinez-Guerra B.A., Garcia-Couturier R., Xancal-Salvador L.F., Esteban-Kenel V., Jaimes-Aquino R.A., Mendoza-Rojas M., Cervantes-Sánchez A., Méndez-Ramos S., Alonso-Montoya J.E. (2023). Predictors of Mortality in Patients with Infections Due to Carbapenem-Resistant Gram-Negative Bacteria. Antibiotics.

[10] Zheng G., Wang S., Lv H., Zhang G. (2022). Nomogram Analysis of Clinical Characteristics and Mortality Risk Factor of Non-Fermentative Gram-Negative Bacteria-Induced Post-Neurosurgical Meningitis. Infect. Drug Resist..

[11] Jean S.-S., Gould I.M., Lee W.-S., Hsueh P.-R. (2019). International Society of Antimicrobial Chemotherapy (ISAC) New Drugs for Multidrug-Resistant Gram-Negative Organisms: Time for Stewardship. Drugs.

[12] Chumbita M., Monzo-Gallo P., Lopera-Mármol C., Aiello T.F., Puerta-Alcalde P., Garcia-Vidal C. (2022). New Treatments for Multidrug-Resistant Non-Fermenting Gram-Negative Bacilli Infections. Rev. Esp. Quimioter..

[13] Giacobbe D.R., Labate L., Russo Artimagnella C., Marelli C., Signori A., Di Pilato V., Aldieri C., Bandera A., Briano F., Cacopardo B. (2024). Use of Cefiderocol in Adult Patients: Descriptive Analysis from a Prospective, Multicenter, Cohort Study. Infect. Dis. Ther..

[14] Hoellinger B., Simand C., Jeannot K., Garijo C., Cristinar M., Reisz F., Danion F., Ursenbach A., Lefebvre N., Boyer P. (2023). Real-World Clinical Outcome of Cefiderocol for Treatment of Multidrug-Resistant Non-Fermenting, Gram Negative Bacilli Infections: A Case Series. Clin. Microbiol. Infect..

[15] Chawla K., Vishwanath S., Munim F.C. (2013). Nonfermenting Gram-Negative Bacilli Other than Pseudomonas Aeruginosa and Acinetobacter Spp. Causing Respiratory Tract Infections in a Tertiary Care Center. J. Glob. Infect. Dis..

[16] Murray P.R., Rosenthal K.S., Pfaller M.A. (2020). Medical Microbiology.

[17] Procop G.W., Church D.L., Hall G.S., Janda W.M., Koneman E.W., Schreckenberger P., Woods G.L. (2017). Koneman’s Color Atlas and Textbook of Diagnostic Microbiology.

[18] Whistler T., Sangwichian O., Jorakate P., Sawatwong P., Surin U., Piralam B., Thamthitiwat S., Promkong C., Peruski L. (2019). Identification of Gram Negative Non-Fermentative Bacteria: How Hard Can It Be?. PLoS Negl. Trop. Dis..

[19] Wisplinghoff H. (2017). *Pseudomonas* Spp., *Acinetobacter* Spp. and Miscellaneous Gram-Negative Bacilli. Infectious Diseases.

[20] Schoch C.L., Ciufo S., Domrachev M., Hotton C.L., Kannan S., Khovanskaya R., Leipe D., Mcveigh R., O’Neill K., Robbertse B. (2020). NCBI Taxonomy: A Comprehensive Update on Curation, Resources and Tools. Database.

[21] Finlay B.B., Falkow S. (1997). Common Themes in Microbial Pathogenicity Revisited. Microbiol. Mol. Biol. Rev..

[22] Park W.S., Lee J., Na G., Park S., Seo S.-K., Choi J.S., Jung W.-K., Choi I.-W. (2022). Benzyl Isothiocyanate Attenuates Inflammasome Activation in Pseudomonas Aeruginosa LPS-Stimulated THP-1 Cells and Exerts Regulation through the MAPKs/NF-κB Pathway. Int. J. Mol. Sci..

[23] Geremia N., Giovagnorio F., Colpani A., De Vito A., Botan A., Stroffolini G., Toc D.-A., Zerbato V., Principe L., Madeddu G. (2024). Fluoroquinolones and Biofilm: A Narrative Review. Pharmaceuticals.

[24] LiPuma J., Currie B., Peacock S.J., Vandamme P., Jorgensen J.H., Carroll K.C., Funke G., Pfaller M.A. (2015). Burkholderia, Stenotrophomonas, Ralstonia, Cupriavidus, Pandoraea, Brevundimonas, Comamonas, Delftia, and Acidovorax. Manual of Clinical Microbiology.

[25] Farfour E., Roux A., Sage E., Revillet H., Vasse M., Vallée A. (2023). Rarely Encountered Gram-Negative Rods and Lung Transplant Recipients: A Narrative Review. Microorganisms.

[26] Sfeir M.M. (2020). Antimicrobial Susceptibility Testing for Glucose-Nonfermenting Gram-Negative Bacteria: The Tip of the Iceberg. Antimicrob. Agents Chemother..

[27] Eucast: EUCAST on “Intrinsic Resistance and Unusual Phenotypes” Updated. https://www.eucast.org/eucast_news/news_singleview?tx_ttnews%5Btt_news%5D=450&cHash=d75c16db85ee0d4369a4148e463d72a8.

[28] Amoureux L., Bador J., Bounoua Zouak F., Chapuis A., de Curraize C., Neuwirth C. (2016). Distribution of the Species of Achromobacter in a French Cystic Fibrosis Centre and Multilocus Sequence Typing Analysis Reveal the Predominance of A. Xylosoxidans and Clonal Relationships between Some Clinical and Environmental Isolates. J. Cyst. Fibros..

[29] Coward A., Kenna D.T.D., Woodford N., Turton J.F., Members of the UK CF Surveillance Working Group (2020). The UK CF Surveillance Working Group comprised Structured Surveillance of Achromobacter, Pandoraea and Ralstonia Species from Patients in England with Cystic Fibrosis. J. Cyst. Fibros..

[30] Spilker T., Vandamme P., LiPuma J.J. (2013). Identification and Distribution of Achromobacter Species in Cystic Fibrosis. J. Cyst. Fibros..

[31] Neidhöfer C., Berens C., Parčina M. (2022). An 18-Year Dataset on the Clinical Incidence and MICs to Antibiotics of Achromobacter Spp. (Labeled Biochemically or by MAL-DI-TOF MS as A. Xylosoxidans), Largely in Patient Groups Other than Those with CF. Antibiotics.

[32] Isler B., Kidd T.J., Stewart A.G., Harris P., Paterson D.L. (2020). Achromobacter Infections and Treatment Options. Antimicrob. Agents Chemother..

[33] Papalia M., Figueroa-Espinosa R., Steffanowski C., Barberis C., Almuzara M., Barrios R., Vay C., Gutkind G., Di Conza J., Radice M. (2020). Expansion and Improvement of MALDI-TOF MS Databases for Accurate Identification of Achromobacter Species. J. Microbiol. Methods.

[34] Gómez-Cerezo J., Suárez I., Ríos J.J., Peña P., García de Miguel M.J., de José M., Monteagudo O., Linares P., Barbado-Cano A., Vázquez J.J. (2003). Achromobacter Xylosoxidans Bacteremia: A 10-Year Analysis of 54 Cases. Eur. J. Clin. Microbiol. Infect. Dis..

[35] Yoon S.H., Kim H., Lim S.M., Kang J.-M. (2022). Nosocomial Outbreak of Achromobacter Spp. Bacteremia Due to Germicide Contamination: A Systematic Review. Eur. Rev. Med. Pharmacol. Sci..

[36] Marion-Sanchez K., Pailla K., Olive C., Le Coutour X., Derancourt C. (2019). Achromobacter Spp. Healthcare Associated Infections in the French West Indies: A Longitudinal Study from 2006 to 2016. BMC Infect Dis.

[37] Tena D., González-Praetorius A., Pérez-Balsalobre M., Sancho O., Bisquert J. (2008). Urinary Tract Infection Due to Achromobacter Xylosoxidans: Report of 9 Cases. Scand. J. Infect. Dis..

[38] Tena D., Martínez N.M., Losa C., Solís S. (2014). Skin and Soft Tissue Infection Caused by Achromobacter Xylosoxidans: Report of 14 Cases. Scand. J. Infect. Dis..

[39] Arshad J.I., Saud A., White D.E., Afshari N.A., Sayegh R.R. (2020). Chronic Conjunctivitis From a Retained Contact Lens. Eye Contact Lens.

[40] Xia R., Otto C., Zeng J., Momeni-Boroujeni A., Kagan J., Meleney K., Libien J. (2018). Achromobacter Endocarditis in Native Cardiac Valves—An Autopsy Case Report and Review of the Literature. Cardiovasc. Pathol..

[41] Veschetti L., Boaretti M., Saitta G.M., Passarelli Mantovani R., Lleò M.M., Sandri A., Malerba G. (2022). Achromobacter Spp. Prevalence and Adaptation in Cystic Fibrosis Lung Infection. Microbiol. Res..

[42] Liu C., Pan F., Guo J., Yan W., Jin Y., Liu C., Qin L., Fang X. (2016). Hospital Acquired Pneumonia Due to Achromobacter Spp. in a Geriatric Ward in China: Clinical Characteristic, Genome Variability, Biofilm Production, Antibiotic Resistance and Integron in Isolated Strains. Front. Microbiol..

[43] Olbrecht M., Echahidi F., Piérard D., Peeters C., Vandamme P., Wybo I., Demuyser T. (2023). In Vitro Susceptibility of Achromobacter Species Isolated from Cystic Fibrosis Patients: A 6-Year Survey. Antimicrob. Agents Chemother..

[44] Bador J., Amoureux L., Blanc E., Neuwirth C. (2013). Innate Aminoglycoside Resistance of Achromobacter Xylosoxidans Is Due to AxyXY-OprZ, an RND-Type Multidrug Efflux Pump. Antimicrob. Agents Chemother..

[45] Yamamoto M., Nagao M., Hotta G., Matsumura Y., Matsushima A., Ito Y., Takakura S., Ichiyama S. (2012). Molecular Characterization of IMP-Type Metallo-β-Lactamases among Multidrug-Resistant Achromobacter Xylosoxidans. J. Antimicrob. Chemother..

[46] El Salabi A., Borra P.S., Toleman M.A., Samuelsen Ø., Walsh T.R. (2012). Genetic and Biochemical Characterization of a Novel Metallo-β-Lactamase, TMB-1, from an Achromobacter Xylosoxidans Strain Isolated in Tripoli, Libya. Antimicrob. Agents Chemother..

[47] Eucast: Clinical Breakpoints and Dosing of Antibiotics. https://www.eucast.org/clinical_breakpoints.

[48] CLSI eClipse Ultimate Access—Powered by Edaptive Technologies. https://em100.edaptivedocs.net/Login.aspx?_ga=2.174075298.1086753664.1709112633-1991978545.1705003341.

[49] Beauruelle C., Lamoureux C., Mashi A., Ramel S., Le Bihan J., Ropars T., Dirou A., Banerjee A., Tandé D., Le Bars H. (2021). In Vitro Activity of 22 Antibiotics against Achromobacter Isolates from People with Cystic Fibrosis. Are There New Therapeutic Options?. Microorganisms.

[50] Denton M., Kerr K.G. (1998). Microbiological and Clinical Aspects of Infection Associated with Stenotrophomonas Maltophilia. Clin. Microbiol. Rev..

[51] Huang C. (2020). Extensively Drug-Resistant Alcaligenes Faecalis Infection. BMC Infect. Dis..

[52] Zorina A.S., Maksimova Y.G., Demakov V.A. (2019). Biofilm Formation by Monocultures and Mixed Cultures of Alcaligenes Faecalis 2 and Rhodococcus Ruber Gt 1. Microbiology.

[53] Spernovasilis N., Ierodiakonou D., Milioni A., Markaki L., Kofteridis D.P., Tsioutis C. (2020). Assessing the Knowledge, Attitudes and Perceptions of Junior Doctors on Antimicrobial Use and Antimicrobial Resistance in Greece. J. Glob. Antimicrob. Resist..

[54] Mishra A., Aggarwal A., Khan F. (2024). Medical Device-Associated Infections Caused by Biofilm-Forming Microbial Pathogens and Controlling Strategies. Antibiotics.

[55] Marino A., Pulvirenti S., Campanella E., Stracquadanio S., Ceccarelli M., Micali C., Tina L.G., Di Dio G., Stefani S., Cacopardo B. (2023). Ceftazidime-Avibactam Treatment for Klebsiella Pneumoniae Bacteremia in Preterm Infants in NICU: A Clinical Experience. Antibiotics.

[56] Marino A., Campanella E., Stracquadanio S., Calvo M., Migliorisi G., Nicolosi A., Cosentino F., Marletta S., Spampinato S., Prestifilippo P. (2023). Ceftazidime/Avibactam and Meropenem/Vaborbactam for the Management of Enterobacterales Infections: A Narrative Review, Clinical Considerations, and Expert Opinion. Antibiotics.

[57] Devanga Ragupathi N.K., Veeraraghavan B. (2019). Accurate Identification and Epidemiological Characterization of Burkholderia Cepacia Complex: An Update. Ann. Clin. Microbiol. Antimicrob..

[58] Yabuuchi E., Kosako Y., Oyaizu H., Yano I., Hotta H., Hashimoto Y., Ezaki T., Arakawa M. (1992). Proposal of Burkholderia Gen. Nov. and Transfer of Seven Species of the Genus Pseudomonas Homology Group II to the New Genus, with the Type Species Burkholderia Cepacia (Palleroni and Holmes 1981) Comb. Nov. Microbiol. Immunol..

[59] Rhodes K.A., Schweizer H.P. (2016). Antibiotic Resistance in Burkholderia Species. Drug Resist. Updat..

[60] Kalish L.A., Waltz D.A., Dovey M., Potter-Bynoe G., McAdam A.J., LiPuma J.J., Gerard C., Goldmann D. (2006). Impact of Burkholderia Dolosa on Lung Function and Survival in Cystic Fibrosis. Am. J. Respir. Crit. Care Med..

[61] Zahariadis G., Levy M.H., Burns J.L. (2003). Cepacia-Like Syndrome Caused by Burkholderia Mutivorans. Can. J. Infect. Dis. Med. Microbiol..

[62] Behroozian S., Zlosnik J.E.A., Xu W., Li L.Y., Davies J.E. (2023). Antibacterial Activity of a Natural Clay Mineral against Burkholderia Cepacia Complex and Other Bacterial Pathogens Isolated from People with Cystic Fibrosis. Microorganisms.

[63] Blanchard A.C., Waters V.J. (2019). Microbiology of Cystic Fibrosis Airway Disease. Semin. Respir. Crit. Care Med..

[64] Nye C., Duckers J., Dhillon R. (2022). Cefiderocol to Manage Chronic, Multi-Drug-Resistant Burkholderia Cepacia Complex Infection in a Patient with Cystic Fibrosis: A Case Report. Access Microbiol..

[65] Akkerman-Nijland A.M., Rottier B.L., Holstein J., Winter R.L.J., Touw D.J., Akkerman O.W., Koppelman G.H. (2023). Eradication of Burkholderia Cepacia Complex in Cystic Fibrosis Patients with Inhalation of Amiloride and Tobramycin Combined with Oral Cotrimoxazole. ERJ Open Res..

[66] Len O., Los-Arcos I., Aguado J.M., Blanes M., Bodro M., Carratalà J., Cordero E., Fariñas M.C., Fernández-Ruiz M., Fortún J. (2020). Selection Criteria of Solid Organ Donors in Relation to Infectious Diseases: A Spanish Consensus. Transplant. Rev..

[67] Mahenthiralingam E., Vandamme P. (2005). Taxonomy and Pathogenesis of the Burkholderia Cepacia Complex. Chron. Respir. Dis..

[68] Isles A., Maclusky I., Corey M., Gold R., Prober C., Fleming P., Levison H. (1984). Pseudomonas Cepacia Infection in Cystic Fibrosis: An Emerging Problem. J. Pediatr..

[69] Sfeir M.M. (2018). Burkholderia Cepacia Complex Infections: More Complex than the Bacterium Name Suggest. J. Infect..

[70] Gutiérrez Santana J.C., Coria Jiménez V.R. (2024). Burkholderia Cepacia Complex in Cystic Fibrosis: Critical Gaps in Diagnosis and Therapy. Ann. Med..

[71] Mahenthiralingam E., Campbell M., Speert D.P. (1995). Burkholderia Cepacia in Cystic Fibrosis. N. Engl. J. Med..

[72] Tseng S.-P., Tsai W.-C., Liang C.-Y., Lin Y.-S., Huang J.-W., Chang C.-Y., Tyan Y.-C., Lu P.-L. (2014). The Contribution of Antibiotic Resistance Mechanisms in Clinical Burkholderia Cepacia Complex Isolates: An Emphasis on Efflux Pump Activity. PLoS ONE.

[73] Abbott F.K., Milne K.E.N., Stead D.A., Gould I.M. (2016). Combination Antimicrobial Susceptibility Testing of Burkholderia Cepacia Complex: Significance of Species. Int. J. Antimicrob. Agents.

[74] Papp-Wallace K.M., Becka S.A., Zeiser E.T., Ohuchi N., Mojica M.F., Gatta J.A., Falleni M., Tosi D., Borghi E., Winkler M.L. (2017). Overcoming an Extremely Drug Resistant (XDR) Pathogen: Avibactam Restores Susceptibility to Ceftazidime for Burkholderia Cepacia Complex Isolates from Cystic Fibrosis Patients. ACS Infect. Dis..

[75] Sutcliffe J.A., O’Brien W., Fyfe C., Grossman T.H. (2013). Antibacterial Activity of Eravacycline (TP-434), a Novel Fluorocycline, against Hospital and Community Pathogens. Antimicrob. Agents Chemother..

[76] Van Dalem A., Herpol M., Echahidi F., Peeters C., Wybo I., De Wachter E., Vandamme P., Piérard D. (2018). In Vitro Susceptibility of Burkholderia Cepacia Complex Isolated from Cystic Fibrosis Patients to Ceftazidime-Avibactam and Ceftolozane-Tazobactam. Antimicrob. Agents Chemother..

[77] Shi H., Chen X., Chen L., Zhu B., Yan W., Ma X. (2023). Burkholderia Cepacia Infection in Children without Cystic Fibrosis: A Clinical Analysis of 50 Cases. Front. Pediatr..

[78] Yamano Y. (2019). In Vitro Activity of Cefiderocol Against a Broad Range of Clinically Important Gram-Negative Bacteria. Clin. Infect. Dis..

[79] Rolston K.V.I., Gerges B., Shelburne S., Aitken S.L., Raad I., Prince R.A. (2020). Activity of Cefiderocol and Comparators against Isolates from Cancer Patients. Antimicrob. Agents Chemother..

[80] Farfour E., d’Epenoux L.R., Muggeo A., Alauzet C., Crémet L., Moussalih S., Roux A., de Verdière S.C., Bosphore A., Corvec S. (2023). In Vitro Susceptibility of Nonfermenting Gram-Negative Rods To Meropenem–Vaborbactam and Delafloxacin. Future Microbiol..

[81] McCarthy B., Casey D., Devane D., Murphy K., Murphy E., Lacasse Y. (2015). Pulmonary Rehabilitation for Chronic Obstructive Pulmonary Disease. Cochrane Database Syst. Rev..

[82] Burton J.K., Craig L.E., Yong S.Q., Siddiqi N., Teale E.A., Woodhouse R., Barugh A.J., Shepherd A.M., Brunton A., Freeman S.C. (2021). Non-Pharmacological Interventions for Preventing Delirium in Hospitalised Non-ICU Patients. Cochrane Database Syst. Rev..

[83] Haidar G., Chan B.K., Cho S.-T., Hughes Kramer K., Nordstrom H.R., Wallace N.R., Stellfox M.E., Holland M., Kline E.G., Kozar J.M. (2023). Phage Therapy in a Lung Transplant Recipient with Cystic Fibrosis Infected with Multidrug-Resistant Burkholderia Multivorans. Transpl. Infect. Dis..

[84] Narayanaswamy V.P., Giatpaiboon S., Baker S.M., Wiesmann W.P., LiPuma J.J., Townsend S.M. (2017). Novel Glycopolymer Sensitizes Burkholderia Cepacia Complex Isolates from Cystic Fibrosis Patients to Tobramycin and Meropenem. PLoS ONE.

[85] Maydaniuk D., Wu B., Truong D., Liyanage S.H., Hogan A.M., Yap Z.L., Yan M., Cardona S.T. (2021). New Auranofin Analogs with Antibacterial Properties against Burkholderia Clinical Isolates. Antibiotics.

[86] Peacock S.J., Schweizer H.P., Dance D.A.B., Smith T.L., Gee J.E., Wuthiekanun V., DeShazer D., Steinmetz I., Tan P., Currie B.J. (2008). Management of Accidental Laboratory Exposure to Burkholderia Pseudomallei and B. Mallei. Emerg. Infect. Dis. J..

[87] Cheng A.C., Dance D.a.B., Currie B.J. (2005). Bioterrorism, Glanders and Melioidosis. Eurosurveillance.

[88] Schmoock G., Elschner M., Sprague L.D. (2015). Clear Distinction between Burkholderia Mallei and Burkholderia Pseudomallei Using Fluorescent motB Primers. Acta Vet. Scand..

[89] Gassiep I., Armstrong M., Norton R. (2020). Human Melioidosis. Clin. Microbiol. Rev..

[90] Chakravorty A., Heath C. (2019). Melioidosis: An Updated Review. Aust. J. Gen. Pract..

[91] Wiersinga W.J., Currie B.J., Peacock S.J. (2012). Melioidosis. N. Engl. J. Med..

[92] Cheng A.C., Currie B.J. (2005). Melioidosis: Epidemiology, Pathophysiology, and Management. Clin. Microbiol. Rev..

[93] Wiersinga W.J., Virk H.S., Torres A.G., Currie B.J., Peacock S.J., Dance D.A.B., Limmathurotsakul D. (2018). Melioidosis. Nat. Rev. Dis. Primers.

[94] Karunanayake P. (2022). Melioidosis: Clinical Aspects. Clin. Med..

[95] Hatcher C.L., Muruato L.A., Torres A.G. (2015). Recent Advances in Burkholderia Mallei and B. Pseudomallei Research. Curr. Trop. Med. Rep..

[96] Dance D.A.B., Wuthiekanun V., Chaowagul W., White N.J. (1989). The Antimicrobial Susceptibility of Pseudomonas Pseudomallei. Emergence of Resistance in Vitro and during Treatment. J. Antimicrob. Chemother..

[97] Jenney A.W.J., Lum G., Fisher D.A., Currie B.J. (2001). Antibiotic Susceptibility of *Burkholderia Pseudomallei* from Tropical Northern Australia and Implications for Therapy of Melioidosis. Int. J. Antimicrob. Agents.

[98] Currie B.J. (2015). Melioidosis: Evolving Concepts in Epidemiology, Pathogenesis, and Treatment. Semin. Respir. Crit. Care Med..

[99] Lipsitz R., Garges S., Aurigemma R., Baccam P., Blaney D.D., Cheng A.C., Currie B.J., Dance D., Gee J.E., Larsen J. (2012). Workshop on Treatment of and Postexposure Prophylaxis for Burkholderia Pseudomallei and B. Mallei Infection, 2010. Emerg. Infect. Dis..

[100] Pitman M.C., Luck T., Marshall C.S., Anstey N.M., Ward L., Currie B.J. (2015). Intravenous Therapy Duration and Outcomes in Melioidosis: A New Treatment Paradigm. PLoS Neglected Trop. Dis..

[101] Van Zandt K.E., Greer M.T., Gelhaus H.C. (2013). Glanders: An Overview of Infection in Humans. Orphanet J. Rare Dis..

[102] Dvorak G.D., Spickler A.R. (2008). Glanders. J. Am. Vet. Med. Assoc..

[103] Burnard D., Robertson G., Henderson A., Falconer C., Bauer M.J., Cottrell K., Gassiep I., Norton R., Paterson D.L., Harris P.N.A. (2021). Burkholderia Pseudomallei Clinical Isolates Are Highly Susceptible In Vitro to Cefiderocol, a Siderophore Cephalosporin. Antimicrob. Agents Chemother..

[104] Tapia D., Sanchez-Villamil J.I., Torres A.G. (2019). Emerging Role of Biologics for the Treatment of Melioidosis and Glanders. Expert. Opin. Biol. Ther..

[105] Eickhoff T.C., Bennett J.V., Hayes P.S., Feeley J. (1970). Pseudomonas Pseudomallei: Susceptibility to Chemotherapeutic Agents. J. Infect. Dis..

[106] Chan Y.Y., Tan T.M.C., Ong Y.M., Chua K.L. (2004). BpeAB-OprB, a Multidrug Efflux Pump in Burkholderia Pseudomallei. Antimicrob. Agents Chemother..

[107] Moore R.A., DeShazer D., Reckseidler S., Weissman A., Woods D.E. (1999). Efflux-Mediated Aminoglycoside and Macrolide Resistance in Burkholderia Pseudomallei. Antimicrob. Agents Chemother..

[108] Anuntagool N., Wuthiekanun V., White N.J., Currie B.J., Sermswan R.W., Wongratanacheewin S., Taweechaisupapong S., Chaiyaroj S.C., Sirisinha S. (2006). LIPOPOLYSACCHARIDE HETEROGENEITY AMONG BURKHOLDERIA PSEUDOMALLEI FROM DIFFERENT GEOGRAPHIC AND CLINICAL ORIGINS. Am. J. Trop. Med. Hyg..

[109] Sawasdidoln C., Taweechaisupapong S., Sermswan R.W., Tattawasart U., Tungpradabkul S., Wongratanacheewin S. (2010). Growing Burkholderia Pseudomallei in Biofilm Stimulating Conditions Significantly Induces Antimicrobial Resistance. PLoS ONE.

[110] Zemanick E.T., Hoffman L.R. (2016). Cystic Fibrosis: Microbiology and Host Response. Pediatr. Clin. N. Am..

[111] Gruzelle V., Guet-Revillet H., Segonds C., Bui S., Macey J., Chiron R., Michelet M., Murris-Espin M., Mittaine M. (2020). Management of Initial Colonisations with Burkholderia Species in France, with Retrospective Analysis in Five Cystic Fibrosis Centres: A Pilot Study. BMC Pulm. Med..

[112] King E.O. (1959). Studies on a Group of Previously Unclassified Bacteria Associated with Meningitis in Infants. Am. J. Clin. Pathol..

[113] Lin J.-N., Lai C.-H., Yang C.-H., Huang Y.-H. (2019). Elizabethkingia Infections in Humans: From Genomics to Clinics. Microorganisms.

[114] Dziuban E.J., Franks J.L., So M., Peacock G., Blaney D.D. (2018). Elizabethkingia in Children: A Comprehensive Review of Symptomatic Cases Reported From 1944 to 2017. Clin. Infect. Dis..

[115] Zajmi A., Teo J., Yeo C.C. (2022). Epidemiology and Characteristics of Elizabethkingia Spp. Infections in Southeast Asia. Microorganisms.

[116] Kämpfer P., Matthews H., Glaeser S.P., Martin K., Lodders N., Faye I. (2011). Elizabethkingia Anophelis Sp. Nov., Isolated from the Midgut of the Mosquito Anopheles Gambiae. Int. J. Syst. Evol. Microbiol..

[117] Hem S., Jarocki V.M., Baker D.J., Charles I.G., Drigo B., Aucote S., Donner E., Burnard D., Bauer M.J., Harris P.N.A. (2021). Genomic Analysis of Elizabethkingia Species from Aquatic Environments: Evidence for Potential Clinical Transmission. Curr. Res. Microb. Sci..

[118] Hwang J.-H., Kim J., Kim J., Mo S. (2021). Elizabethkingia Argenteiflava Sp. Nov., Isolated from the Pod of Soybean, Glycine Max. Int. J. Syst. Evol. Microbiol..

[119] Perrin A., Larsonneur E., Nicholson A.C., Edwards D.J., Gundlach K.M., Whitney A.M., Gulvik C.A., Bell M.E., Rendueles O., Cury J. (2017). Evolutionary Dynamics and Genomic Features of the Elizabethkingia Anophelis 2015 to 2016 Wisconsin Outbreak Strain. Nat Commun.

[120] Guerpillon B., Fangous M.S., Le Breton E., Artus M., le Gall F., Khatchatourian L., Talarmin J.P., Plesiat P., Jeannot K., Saidani N. (2022). Elizabethkingia Anophelis Outbreak in France. Infect. Dis. Now..

[121] Lau S.K.P., Chow W.-N., Foo C.-H., Curreem S.O.T., Lo G.C.-S., Teng J.L.L., Chen J.H.K., Ng R.H.Y., Wu A.K.L., Cheung I.Y.Y. (2016). Elizabethkingia Anophelis Bacteremia Is Associated with Clinically Significant Infections and High Mortality. Sci. Rep..

[122] Hoque S.N., Graham J., Kaufmann M.E., Tabaqchali S. (2001). Chryseobacterium (Flavobacterium) Meningosepticum Outbreak Associated with Colonization of Water Taps in a Neonatal Intensive Care Unit. J. Hosp. Infect..

[123] Comba I.Y., Schuetz A.N., Misra A., Friedman D.Z.P., Stevens R., Patel R., Lancaster Z.D., Shah A. (2022). Antimicrobial Susceptibility of Elizabethkingia Species: Report from a Reference Laboratory. J. Clin. Microbiol..

[124] Wu C., Xiong L., Liao Q., Zhang W., Xiao Y., Xie Y. (2024). Clinical Manifestations, Antimicrobial Resistance and Genomic Feature Analysis of Multidrug-Resistant Elizabethkingia Strains. Ann. Clin. Microbiol. Antimicrob..

[125] Hu S., Lv Y., Xu H., Zheng B., Xiao Y. (2022). Biofilm Formation and Antibiotic Sensitivity in Elizabethkingia Anophelis. Front. Cell. Infect. Microbiol..

[126] Huang Y.-S., Chuang Y.-C., Chen P.-Y., Chou P.-C., Wang J.-T. (2024). In Vitro Activity of Cefiderocol and Comparator Antibiotics against Multidrug-Resistant Non-Fermenting Gram-Negative Bacilli. JAC Antimicrob. Resist..

[127] Puah S.M., Fong S.P., Kee B.P., Puthucheary S.D., Chua K.H. (2022). Molecular Identification and Biofilm-Forming Ability of Elizabethkingia Species. Microb. Pathog..

[128] Verduin C.M., Hol C., Fleer A., van Dijk H., van Belkum A. (2002). *Moraxella Catarrhalis*: From Emerging to Established Pathogen. Clin. Microbiol. Rev..

[129] Ho K.-T., Su K.-W., Liao S.-L., Chiu C.-Y., Hua M.-C., Huang J.-L., Huang Y.-C., Chiu C.-H., Lin T.-Y., Tsai M.-H. (2023). Longitudinal Investigation of Pathogenic Bacterial Colonization in Early Childhood: Emphasis on the Determinants of *Moraxella Catarrhalis* Colonization. J. Microbiol. Immunol. Infect..

[130] Verhaegh S.J.C., Snippe M.L., Levy F., Verbrugh H.A., Jaddoe V.W.V., Hofman A., Moll H.A., van Belkum A., Hays J.P. (2011). Colonization of Healthy Children by Moraxella Catarrhalis Is Characterized by Genotype Heterogeneity, Virulence Gene Diversity and Co-Colonization with Haemophilus Influenzae. Microbiology.

[131] Sillanpää S., Oikarinen S., Sipilä M., Kramna L., Rautiainen M., Huhtala H., Aittoniemi J., Laranne J., Hyöty H., Cinek O. (2016). Moraxella Catarrhalis Might Be More Common than Expected in Acute Otitis Media in Young Finnish Children. J. Clin. Microbiol..

[132] Fox-Lewis A., Coltart G., Rice S., Sen R., Gourtsoyannis Y., Hyare H., Gupta R.K. (2016). Extensive Subclinical Sinusitis Leading to *Moraxella Osloensis* Meningitis. IDCases.

[133] Murphy T.F., Brauer A.L., Grant B.J.B., Sethi S. (2005). Moraxella Catarrhalis in Chronic Obstructive Pulmonary Disease. Am. J. Respir. Crit. Care Med..

[134] Karalus R., Campagnari A. (2000). Moraxella Catarrhalis: A Review of an Important Human Mucosal Pathogen. Microbes Infect..

[135] Koleri J., Petkar H.M., Husain A.A.M., Almaslamani M.A., Omrani A.S. (2022). *Moraxella Osloensis* Bacteremia, a Case Series and Review of the Literature. IDCases.

[136] Sawada N., Morohashi T., Mutoh T., Kuwana T., Yamaguchi J., Kinoshita K., Morioka I., Hao H. (2020). Moraxella Lacunata Infection Accompanied by Acute Glomerulonephritis. Open Med..

[137] Zaman S., Greene J. (2021). Moraxella Bacteremia in Cancer Patients. Cureus.

[138] Correa-Martínez C.L., Rauwolf K.K., Schuler F., Füller M., Kampmeier S., Groll A.H. (2019). Moraxella Nonliquefaciens Bloodstream Infection and Sepsis in a Pediatric Cancer Patient: Case Report and Literature Review. BMC Infect. Dis..

[139] Postma G.C., Carfagnini J.C., Minatel L. (2008). Moraxella Bovis Pathogenicity: An Update. Comp. Immunol. Microbiol. Infect. Dis..

[140] Hoban D.J., Doern G.V., Fluit A.C., Roussel-Delvallez M., Jones R.N. (2001). Worldwide Prevalence of Antimicrobial Resistance in Streptococcus Pneumoniae, Haemophilus Influenzae, and Moraxella Catarrhalis in the SENTRY Antimicrobial Surveillance Program, 1997–1999. Clin. Infect. Dis..

[141] Mulu W., Yizengaw E., Alemu M., Mekonnen D., Hailu D., Ketemaw K., Abera B., Kibret M. (2018). Pharyngeal Colonization and Drug Resistance Profiles of Morraxella Catarrrhalis, Streptococcus Pneumoniae, Staphylococcus Aureus, and Haemophilus Influenzae among HIV Infected Children Attending ART Clinic of Felegehiwot Referral Hospital, Ethiopia. PLoS ONE.

[142] Beekmann S.E., Heilmann K.P., Richter S.S., García-de-Lomas J., Doern G.V. (2005). Antimicrobial Resistance in *Streptococcus Pneumoniae*, *Haemophilus Influenzae*, *Moraxella Catarrhalis* and Group A β-Haemolytic Streptococci in 2002–2003. Int. J. Antimicrob. Agents.

[143] Esel D., Ay-Altintop Y., Yagmur G., Gokahmetoglu S., Sumerkan B. (2007). Evaluation of Susceptibility Patterns and BRO β-Lactamase Types among Clinical Isolates of Moraxella Catarrhalis. Clin. Microbiol. Infect..

[144] Kaderabkova N., Bharathwaj M., Furniss R.C.D., Gonzalez D., Palmer T., Mavridou D.A.I. (2022). The Biogenesis of β-Lactamase Enzymes. Microbiology.

[145] Raveendran S., Kumar G., Sivanandan R.N., Dias M. (2020). Moraxella Catarrhalis: A Cause of Concern with Emerging Resistance and Presence of BRO Beta-Lactamase Gene—Report from a Tertiary Care Hospital in South India. Int. J. Microbiol..

[146] Powell M., McVey D., Kassim M.H., Chen H.Y., Williams J.D. (1991). Antimicrobial Susceptibility of Streptococcus Pneumoniae, Haemophilus Influenzae and Moraxella (Branhamella) Catarrhalis Isolated in the UK from Sputa. J. Antimicrob. Chemother..

[147] Bair K.L., Campagnari A.A. (2019). Moraxella Catarrhalis Promotes Stable Polymicrobial Biofilms With the Major Otopathogens. Front. Microbiol..

[148] Armbruster C.E., Hong W., Pang B., Weimer K.E.D., Juneau R.A., Turner J., Swords W.E. (2010). Indirect Pathogenicity of Haemophilus Influenzae and Moraxella Catarrhalis in Polymicrobial Otitis Media Occurs via Interspecies Quorum Signaling. mBio.

[149] Flamm R.K., Sader H.S., Farrell D.J., Jones R.N. (2012). Macrolide and Tetracycline Resistance among *Moraxella Catarrhalis* Isolates from 2009 to 2011. Diagn. Microbiol. Infect. Dis..

[150] Hsu S.-F., Lin Y.-T., Chen T.-L., Siu L.K., Hsueh P.-R., Huang S.-T., Fung C.-P. (2012). Antimicrobial Resistance of *Moraxella Catarrhalis* Isolates in Taiwan. J. Microbiol. Immunol. Infect..

[151] Wallace R.J., Nash D.R., Steingrube V.A. (1990). Antibiotic Susceptibilities and Drug Resistance in Moraxella (Branhamella) Catarrhalis. Am. J. Med..

[152] Roberts M.C., Pang Y.J., Spencer R.C., Winstanley T.G., Brown B.A., Wallace R.J. (1991). Tetracycline Resistance in Moraxella (Branhamella) Catarrhalis: Demonstration of Two Clonal Outbreaks by Using Pulsed-Field Gel Electrophoresis. Antimicrob. Agents Chemother..

[153] Moreno E., Middlebrook E.A., Altamirano-Silva P., Al Dahouk S., Araj G.F., Arce-Gorvel V., Arenas-Gamboa Á., Ariza J., Barquero-Calvo E., Battelli G. (2023). If You’re Not Confused, You’re Not Paying Attention: Ochrobactrum Is Not Brucella. J. Clin. Microbiol..

[154] Kettaneh A., Weill F.-X., Poilane I., Fain O., Thomas M., Herrmann J.-L., Hocqueloux L. (2003). Septic Shock Caused by Ochrobactrum Anthropi in an Otherwise Healthy Host. J. Clin. Microbiol..

[155] Ryan M.P., Pembroke J.T. (2020). The Genus Ochrobactrum as Major Opportunistic Pathogens. Microorganisms.

[156] Rastogi N., Mathur P. (2017). Ochrobactrum Anthropi: An Emerging Pathogen Causing Meningitis with Sepsis in a Neurotrauma Patient. J. Infect. Dev. Ctries..

[157] Thoma B., Straube E., Scholz H.C., Al Dahouk S., Zöller L., Pfeffer M., Neubauer H., Tomaso H. (2009). Identification and Antimicrobial Susceptibilities of *Ochrobactrum* Spp.. Int. J. Med. Microbiol..

[158] Higgins C.S., Avison M.B., Jamieson L., Simm A.M., Bennett P.M., Walsh T.R. (2001). Characterization, Cloning and Sequence Analysis of the Inducible Ochrobactrum Anthropi AmpC β-Lactamase. J. Antimicrob. Chemother..

[159] Shanthini T., Manohar P., Samna S., Srividya R., Bozdogan B., Rameshpathy M., Ramesh N. (2019). Emergence of Plasmid-Borne Blaoxa-181 Gene in Ochrobactrum Intermedium: First Report from India. Access Microbiol..

[160] Chen Y., Liao K., Ai L., Guo P., Huang H., Wu Z., Liu M. (2017). Bacteremia Caused by Bergeyella Zoohelcum in an Infective Endocarditis Patient: Case Report and Review of Literature. BMC Infect. Dis..

[161] Grams T.R., Kim D.Y., McElvania E. (2023). The Brief Case: Bergeyella Zoohelcum Bacteremia in an Immunocompromised 69-Year-Old Patient. J. Clin. Microbiol..

[162] Reina J., Borrell N. (1992). Leg Abscess Caused by Weeksella Zoohelcum Following a Dog Bite. Clin. Infect. Dis..

[163] Montejo M., Aguirrebengoa K., Ugalde J., Lopez L., Nieto J.A.S., Hernández J.L. (2001). Bergeyella Zoohelcum Bacteremia after a Dog Bite. Clin. Infect. Dis..

[164] de la Fuente García Peña L.A., Mendoza García A.U., Villegas-Dominguez J.E., Márquez Celedonio F.G., Arana Vidal H., Azuara Díaz K. (2024). Ventilator-Associated Pneumonia by Weeksella Virosa: Case Report. BMC Infect. Dis..

[165] Manogaran M., Marnejon T., Sarac E. (2004). Pneumonia and Sepsis Due to Weeksella Virosa in an Immunocompromised Patient. Infect. Dis. Clin. Pract..

[166] Vaquera-Aparicio D.N., Mascareñas-De los Santos A.H., Casillas-Vega N., Riojas-Hernández P., Llaca-Díaz J., Herrera-Benavente I., Castillo-Bejarano J.I. (2020). Bacteremia Due to Weeksella Virosa in a Pediatric Patient with Embryonal Rhabdomyosarcoma. Boletín Médico Hosp. Infant. México.

[167] O’Rourke D.P., Rosenbaum M.D., Fox J.G., Anderson L.C., Otto G.M., Pritchett-Corning K.R., Whary M.T. (2015). Chapter 18—Biology and Diseases of Amphibians. Laboratory Animal Medicine.

[168] Jean S.-S., Hsieh T.-C., Ning Y.-Z., Hsueh P.-R. (2017). Role of Vancomycin in the Treatment of Bacteraemia and Meningitis Caused by *Elizabethkingia Meningoseptica*. Int. J. Antimicrob. Agents.

[169] Mukerji R., Kakarala R., Smith S.J., Kusz H.G. (2016). Chryseobacterium Indologenes: An Emerging Infection in the USA. BMJ Case Rep..

[170] Kirby J.T., Sader H.S., Walsh T.R., Jones R.N. (2004). Antimicrobial Susceptibility and Epidemiology of a Worldwide Collection of Chryseobacterium Spp.: Report from the SENTRY Antimicrobial Surveillance Program (1997–2001). J. Clin. Microbiol..

[171] Ryan M.P., Sevjahova L., Gorman R., White S. (2022). The Emergence of the Genus Comamonas as Important Opportunistic Pathogens. Pathogens.

[172] Zhang Y., Li K., Zhan Y., Shi L., Zeng Y., Wang H., Lu Z. (2023). Bacteremia Caused by Comamonas Kerstersii in a Patient with Acute Perforated Appendicitis and Localized Peritonitis: Case Report and Literature Review. Front. Med..

[173] Højgaard S.M.M., Rezahosseini O., Knudsen J.D., Fuglebjerg N.J.U., Skov M., Nielsen S.D., Harboe Z.B. (2022). Characteristics and Outcomes of Patients with Delftia Acidovorans Infections: A Retrospective Cohort Study. Microbiol. Spectr..

[174] Lu T.-L., Huang C. (2024). Retrospective Cohort Study on Delftia Acidovorans Infections in Patients: A Rare and Significant Infection. Infect. Drug Resist..

[175] Orsborne C., Hardy A., Isalska B., Williams S.G., Muldoon E.G. (2014). Acidovorax Oryzae Catheter-Associated Bloodstream Infection. J. Clin. Microbiol..

[176] Farfour E., Vasse M., Vallée A. (2024). Oligella Spp.: A Systematic Review on an Uncommon Urinary Pathogen. Eur. J. Clin. Microbiol. Infect. Dis..

[177] Ziogou A., Giannakodimos A., Giannakodimos I., Tsantes A.G., Ioannou P. (2024). Pandoraea Infections in Humans—A Systematic Review. J. Clin. Med..

[178] Lin C., Luo N., Xu Q., Zhang J., Cai M., Zheng G., Yang P. (2019). Pneumonia Due to Pandoraea Apista after Evacuation of Traumatic Intracranial Hematomas:A Case Report and Literature Review. BMC Infect. Dis..

[179] Kumaria A., Crusz S.A., Lister M., Kirkman M.A., Macarthur D.C. (2022). Psychrobacter Piechaudii Shunt Infection: First Report of Human Infection. Childs Nerv. Syst..

[180] Steyaert S., Peeters C., Wieme A.D., Muyldermans A., Vandoorslaer K., Spilker T., Wybo I., Piérard D., LiPuma J.J., Vandamme P. (2024). Novel *Ralstonia* Species from Human Infections: Improved Matrix-Assisted Laser Desorption/Ionization Time-of-Flight Mass Spectrometry-Based Identification and Analysis of Antimicrobial Resistance Patterns. Microbiol. Spectr..

[181] Green H.D., Bright-Thomas R., Kenna D.T., Turton J.F., Woodford N., Jones A.M. (2017). Ralstonia Infection in Cystic Fibrosis. Epidemiol. Infect..

[182] Tiwari S. (2015). Primary Bacteremia Caused by Rhizobium Radiobacter in Neonate: A Rare Case Report. J. Clin. Diagn. Res. JCDR.

[183] Lai C., Teng L., Hsueh P., Yuan A., Tsai K., Tang J., Tien H. (2004). Clinical and Microbiological Characteristics of *Rhizobium Radiobacter* Infections. Clin. Infect. Dis..

[184] Müller S., Von Bonin S., Schneider R., Krüger M., Quick S., Schröttner P. (2023). Shewanella Putrefaciens, a Rare Human Pathogen: A Review from a Clinical Perspective. Front. Cell. Infect. Microbiol..

[185] Yu K., Huang Z., Xiao Y., Wang D. (2022). *Shewanella* Infection in Humans: Epidemiology, Clinical Features and Pathogenicity. Virulence.

[186] Antonelli A., Di Palo D.M., Galano A., Becciani S., Montagnani C., Pecile P., Galli L., Rossolini G.M. (2015). Intestinal Carriage of Shewanella Xiamenensis Simulating Carriage of OXA-48–Producing Enterobacteriaceae. Diagn. Microbiol. Infect. Dis..

[187] Zong Z. (2011). Nosocomial Peripancreatic Infection Associated with Shewanella Xiamenensis. J. Med. Microbiol..

[188] Holt H.M., Gahrn-Hansen B., Bruun B. (2005). Shewanella Algae and Shewanella Putrefaciens: Clinical and Microbiological Characteristics. Clin. Microbiol. Infect..

[189] Grimaldi D., Doloy A., Fichet J., Bourgeois E., Zuber B., Wajsfisz A., Mira J.P., Poyart C., Pène F. (2012). Necrotizing Fasciitis and Septic Shock Related to the Uncommon Gram-Negative Pathogen Sphingobacterium Multivorum. J. Clin. Microbiol..

[190] Barahona F., Slim J. (2015). Sphingobacterium Multivorum: Case Report and Literature Review. New Microbes New Infect..

[191] Fass R.J., Bamishan J. (1980). In Vitro Susceptibilities of Nonfermentative Gram-Negative Bacilli Other than Pseudomonas Aeruginosa to 32 Antimicrobial Agents. Clin. Infect. Dis..

[192] Blahová J., Králiková K., Krčméry V., Kuboňová K. (1997). Hydrolysis of Imipenem, Meropenem, Ceftazidime, and Cefepime by Multiresistant Nosocomial Strains ofSphingobacterium Multivorum. Eur. J. Clin. Microbiol. Infect. Dis..

